# Ultrasound Guided Arthroscopic Removal of Calcific Tendonitis: A Minimum of 2-Year Followup

**DOI:** 10.3390/jcm12093114

**Published:** 2023-04-25

**Authors:** Syed Mohammed Taif Rizvi, David Qiu, Patrick Lam, Lisa Hackett, Judie Walton, George A. C. Murrell

**Affiliations:** Orthopaedic Research Institute, St George Hospital Campus, University of New South Wales, Sydney, NSW 2217, Australiapatlam.ori@gmail.com (P.L.);

**Keywords:** calcific tendonitis, ultrasound-guided removal, localization, biopsy needle, pain, stiffness

## Abstract

**Background:** We have developed a novel technique for managing rotator cuff calcific tendonitis, involving arthroscopic debridement of calcific tendonitis with localization assistance from a breast biopsy needle under ultrasound guidance. While we have demonstrated encouraging results at six-month follow-up, the medium-term outcomes and the long-term outcomes of this technique at 2 years or beyond are unknown. The aim of this paper was to determine if this technique was successful in resolving symptoms after two years and beyond. **Study Design:** Retrospective Cohort Study. **Methods**: Patients who underwent arthroscopic debridement of calcific tendonitis with localization assistance from a breast biopsy needle under ultrasound guidance by a senior surgeon were evaluated using patient-rated pain scores and functional status with the use of the Likert scales and via examiner-rated shoulder range-of-motion and strength at the pre-operative visit, at 1, 6, 12, and 24 weeks post-operatively, and long-term at a mean of 249 weeks after surgery. **Results:** At a mean follow-up period of 4.8 years (range, 2–10 years), 31 patients (33 shoulders) experienced significant improvement in the severity of pain at rest, with overhead activities, and during sleep compared to their pre-operative presentation (*p* < 0.001). The patient experienced less frequent pain during activities and sleep, and a decreased frequency of extreme pain (*p* < 0.001). Passive range of abduction (*p* = 0.003), forward flexion (*p* < 0.001), and supraspinatus strength (*p* = 0.018) improved compared to the presurgical presentation. Out of 27 patients, 24 patients (89%) had complete resolution of calcific tendonitis, and 26 patients (96%) had an intact rotator cuff. **Conclusion:** Arthroscopic debridement of calcific tendonitis with localization assistance from a breast biopsy needle under ultrasound guidance was very effective. Patients had significant pain relief, improved range of motion, and a reduction in stiffness at a mean post-operative period of 4.8 years. Patients had a significant reduction in residual calcification, and rotator cuff integrity was largely preserved by long-term follow-up. **What is known about this subject:** Calcific tendonitis of the rotator cuff is one of the most painful and debilitating disorders of the shoulder. This condition is characterized by the deposition of calcium-phosphate crystals within the rotator cuff tendons. Arthroscopic debridement and excision of rotator cuff calcifications have proven to be efficacious treatments with regards to clinical and functional outcomes in the short and medium term. Identifying the calcific lesion intra-operatively, however, can prove to be challenging. Furthermore, inadequate excision of the calcific deposit has been shown to have poorer clinical outcomes. We designed a technique that utilizes the assistance of ultrasound to guide a localization-biopsy wire to the calcific lesion. This technique aids in precisely identifying the location of the lesion intra-operatively to optimize accuracy in removing the maximum amount of calcific deposit possible. A short-term follow-up study by us has demonstrated successful outcomes with regards to the return of function and relief of pain. However, there have been no studies evaluating the effectiveness of this particular technique beyond six months. **What this study adds to current knowledge:** At a mean of 4.8 years, arthroscopic debridement of calcific tendonitis, using our technique, was successful in relieving the severity and frequency of pain with overhead activities, pain at rest, and pain during sleep, as well as improving range of motion.

## 1. Introduction

Calcific tendonitis of the rotator cuff is one of the most painful and debilitating disorders of the shoulder. This disorder typically presents with pain aggravated by abduction [1], and tenderness over the supraspinatus insertion [2]. The prevalence of calcific tendonitis in patients with shoulder pain ranges between 6.5% and 42% [3]. This condition commonly arises in sedentary employees between the ages of 30 and 50 [1], and it affects females more frequently than males [3].

The etiology and pathophysiology of calcific tendonitis are undetermined. This condition is characterized by the deposition of calcium-phosphate crystals within the rotator cuff tendons, primarily involving the supraspinatus tendon (80%) [3]. Hackett et al. [4] demonstrated that, compared to patients who suffered rotator cuff tears alone, patients who had associated calcific deposits within their tendon had significantly more pain, inflammation, neo-vascularization, and neo-innervation within the tendon. The tendon was also characterized by a 24-fold increase in pro-inflammatory cytokines.

First-line management of calcific tendonitis is often via non-steroidal anti-inflammatory drugs, corticosteroids, extracorporeal shockwave therapy, and ultrasound-guided percutaneous lavage [1,2,4,5,6,7,8,9,10,11,12,13,14]. For patients with progressive symptoms that are refractory to conservative treatment, arthroscopic debridement and excision of rotator cuff calcifications have proven to be efficacious treatments with regards to clinical and functional outcomes in the short and medium term [15]. Identifying the calcific lesion intra-operatively, however, can prove to be challenging, with one study showing that in 16–18% of arthroscopic cases, no calcific deposit could be located [16]. Furthermore, inadequate excision of the calcific deposit has been shown to have poorer clinical outcomes. Jerosch et al. [16] demonstrated how patients post-arthroscopic excision who had elimination or reduction of their calcium deposits experienced significantly greater post-operative shoulder function (as measured by the constant score) compared to those who had no radiographic change post-operatively of the calcium deposit size. Of note, a well-documented risk of operative excision of calcific lesions is the possibility of developing secondary stiffness, with a reported prevalence of 9–15% [17,18]. The development of post-operative stiffness is thought to be due to residual calcium fragments inciting further inflammation within the subacromial bursa [17,19,20,21].

**Figure 1:**
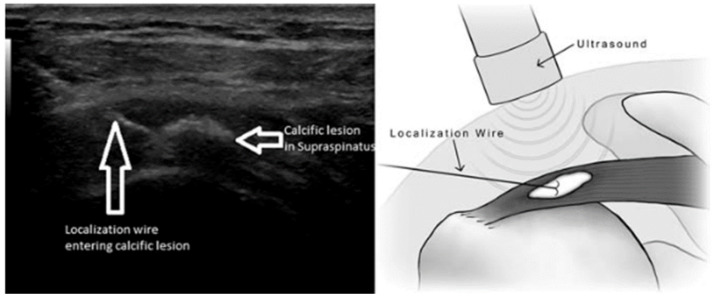
Ultrasound of localization wire entering calcific lesion on left, and illustration on right [22].

We designed a technique that utilizes the assistance of ultrasound to guide a localization-biopsy wire to the calcific lesion. This technique aids in precisely identifying the location of the lesion intra-operatively to optimize accuracy in removing the maximal amount of calcific deposit possible (Figure 1). A short-term follow-up study by us has demonstrated successful outcomes with regards to the return of function and relief of pain [3]. However, there have been no studies evaluating the effectiveness of this particular technique beyond six months. The aim of this study was therefore to evaluate the long-term (minimum 24 months) clinical and functional outcomes of ultrasound-guided arthroscopic debridement of calcific tendonitis.

## 2. Materials and Methods

### 2.1. Study Design

This was a retrospective cohort study. Ethics approval was obtained from the South Eastern Sydney Local Health District Human Research and Ethics Committee (SESLHD). Patients were included in this study if they were diagnosed with calcific tendonitis of the rotator cuff, underwent a primary arthroscopic ultrasound-guided debridement of calcific tendonitis with the assistance of a localization wire (performed by the senior surgeon), and attended a minimum two-year follow-up period.

Patients were excluded from this study if they had (1) prior surgery for calcification; (2) irreparable rotator cuff tear in the affected shoulder; or (3) severe (above grade 3) osteoarthritis in the affected shoulder. The criteria used by our facility for a diagnosis of calcific tendonitis were (1) a history or presentation with shoulder pain and (2) visualization of a calcific lesion on diagnostic ultrasound and/or X-ray imaging.

### 2.2. Operative Technique

Following regional interscalene anesthesia, patients were placed in the beach chair position under intra-venous sedation in preparation for arthroscopy. PorTable 2-D ultrasound (GE Logic E9, Milwaukee, WI, USA and Siemens ACUSON S2000™ ultrasound system (HELIX Evolution ultrasound system; Siemens Medical Solutions^®^, Mountain View, CA, USA)) was used to locate the calcific lesion. After the calcific deposit was identified, the skin around the lateral portal was sterilized with standard betadine-alcohol surgical preparatory solution. Under ultrasound guidance, a breast-biopsy localization wire with its introducer needle (Bard DuaLok, Bard Peripheral Vascular Inc., Tempe, AZ, USA) was entered through the skin and into the subacromial space, and was placed into the calcific deposit (Figure 1). While the introducer needle tip (20-gauge needle) was held, the localization wire was directed forward with two barbs deployed into the lesion to secure the wire within the lesion. The needle was removed, with the localization wire remaining in the calcific deposit and visible on the surface of the skin.

The shoulder and upper limb were then prepped with iodine and alcohol and draped, taking care especially to not dislodge the wire. A clear adhesive surgical preparatory drape (Ioban 2, 3M Inc., St. Paul, MN, USA) was applied to the skin, securing the flexible wire onto the skin.

Diagnostic glenohumeral joint arthroscopy was conducted through a posterior portal to assess intra-articular pathology, and then the arthroscope was moved to the subacromial space. Using a spinal needle, a standard lateral portal was established, and the localization wire was identified and followed, leading towards the location of the calcific deposit. The integrity of the rotator cuff was visually assessed, and then the localization wire was removed. The calcific lesion was then decompressed through the lateral portal via trephination with an 18-gauge spinal needle, with the calcific material usually extruded in a “snowy” pattern. A small (4 mm) diameter motorized shaver (Stryker) was used to further debride the lesion. After removal, the remaining rotator cuff tendon was assessed for defects. Small defects were not repaired, but significant defects, if present, were repaired. Further information regarding this procedure is available in the initial technique paper (a video demonstration of the above procedure is available at http://vumedi.com (accessed on 4 April 2020) [22].

The wound was subsequently closed via 3–0 prolene sutures and dressings, and a sling for comfort was applied. Routine post-operative protocols consisted of range-of-motion exercises and gradually increased shoulder activity [23]. If a rotator cuff repair was performed, the facility’s rotator cuff rehabilitation protocol was followed post-operatively [24].

### 2.3. Pain Assessment

Patients completed a standardized modified L’Insalata shoulder questionnaire at each clinical visit (pre-operatively, and post-operatively at 1, 6, 12, 24 weeks, and at long-term follow-up). On the questionnaire, there were several categories of rating: the frequency of pain during activity, at sleep, and at extreme levels; the level of pain at rest, during overhead activities and when sleeping; level of shoulder stiffness; difficulty in reaching behind the back and overhead; overall shoulder rating; current level of activity at work and current level of sport, based on Likert scales [24].

Patient answers were converted to the numbers in the brackets as seen for further statistical analysis. Patients responded to questions concerning the frequency of pain with never (0), sometimes (1), monthly (2), weekly (3), and all the time (4). The responses to questions the about level of pain were ranked from none (0), mild (1), moderate (2), severe (3) and very severe (4). The level of overall shoulder stiffness was categorized as not at all (0), a little (1), moderately (2), quite (3) or very (4). The categories for questions pertaining to difficulty with activities reaching behind their head and overhead were none (0), mild (1), moderate (2), severe (3) or very severe (4). Patients ranked their shoulder status as good (4), fair (3), poor (2), bad (1) or very bad (0). The level of activity at work and level of sport being played were separated into none (0), light (1), moderate (2) and strenuous (3); and none (0), hobby (1), club (2) and national (3), respectively.

### 2.4. Range of Motion and Strength Assessment

Shoulder range of motion and strength were also tested in this study by trained examiners. Shoulder passive range of motion was assessed in the following movements: forward flexion, abduction, external rotation, and internal rotation. Passive range of motion was performed by visual estimation with this technique shown previously to have fair to good reliability [25]. The angles of forward flexion, abduction, and external rotation were recorded in degrees. The measurement for internal rotation was defined as the highest vertebral level the tip of the thumb could reach when the patient reached behind their back.

Shoulder strength was tested in internal rotation, external rotation, empty can position (supraspinatus), lift-off test, and adduction. Strength was measured utilizing handheld dynamometers, which were shown to be an objective method to measure shoulder strength, with high inter-rater and intra-rater reliability [26,27,28]. Measurements were obtained for all of the maximal loads in Newtons when the patients were instructed to build a maximum voluntary contraction for 1–2 s prior to the application by resistance of the examiner to the action. They were then instructed to hold the action for 4–5 s after resistance was applied.

### 2.5. Statistical Analysis

Statistical analysis was conducted using Microsoft Excel and SPSS Statistics (IBM Corp., Armonk, NY, USA). Multiple paired T-tests were utilized for parametric data, data involving range of motion and strength assessment. Wilcoxon signed-rank tests were utilized for non-parametric data, data involving pain assessment, and comparison of pre-operative scores versus post-operative scores of the same individuals within this longitudinal study. All results were reported as mean ± standard error of the mean. Statistical significance was set at *p* ≤ 0.05.

### 2.6. Outcomes

The primary outcome was the impact of this procedure on patient-reported severity and frequency of pain (including pain at rest, pain with overhead activity, and pain while sleeping) at minimum two years from surgery, compared to pre-operatively. Secondary outcomes were the examiner-determined shoulder range of motion and strength (measured at 1, 6, 12, 26 weeks, and two or more years post-operation), stiffness, presence of calcium deposits, and whether there were any re-tears in the rotator cuff tendon that underwent calcific removal.

## 3. Results

### 3.1. Study Group

During the study period, the senior surgeon performed 37 cases of ultrasound-guided breast biopsy wire-assisted arthroscopic debridement of calcific tendonitis in 35 patients. Four patients were not included in this medium-long-term follow-up study, as they were lost to follow-up. The study sample therefore consisted of 33 shoulders (of 31 patients). The population group was followed up at a mean of 4.8 years post-debridement with a range of 2 to 10 years. The mean age of the study group was 54 years, with a range of 38 to 76. The study group was comprised of 10 males and 21 females. There were 18 right shoulders and 15 left shoulders assessed in this study (Table 1).

### 3.2. Patient-Ranked Pain

**Figure 2:**
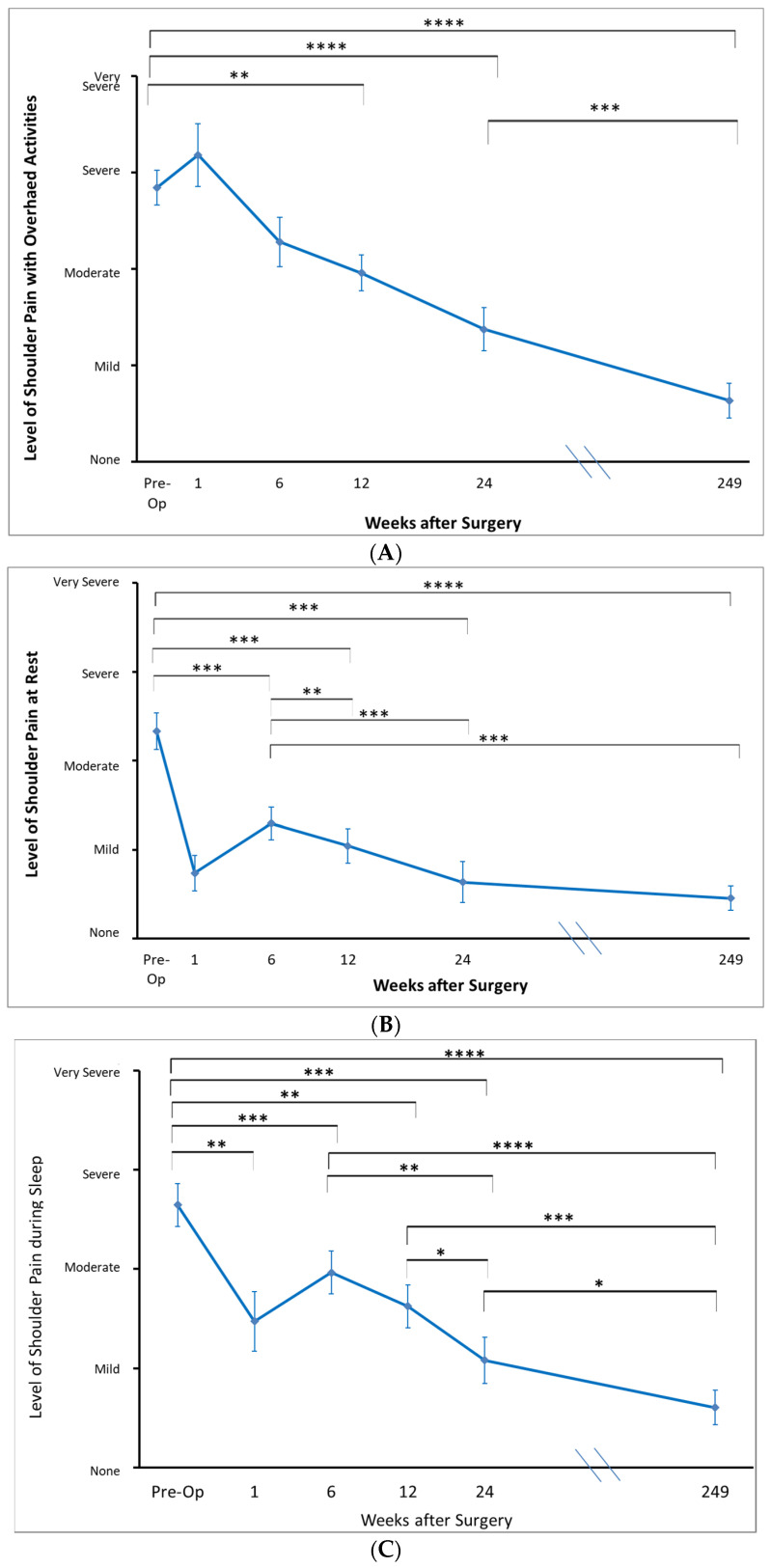
Patient-determined severity of pain, including with overhead activities (**A**), at rest (**B**), and during sleep (**C**). * *p* < 0.05. ** *p* < 0.01. *** *p* < 0.001. **** *p* < 0.0001.

Prior to surgery, patients in this cohort, on average, rated their pain with overhead activities as ‘moderate’ to ‘very severe’ and their pain at rest and overnight as ‘mild’ to ‘very severe’ (Figure 2).

**Figure 3:**
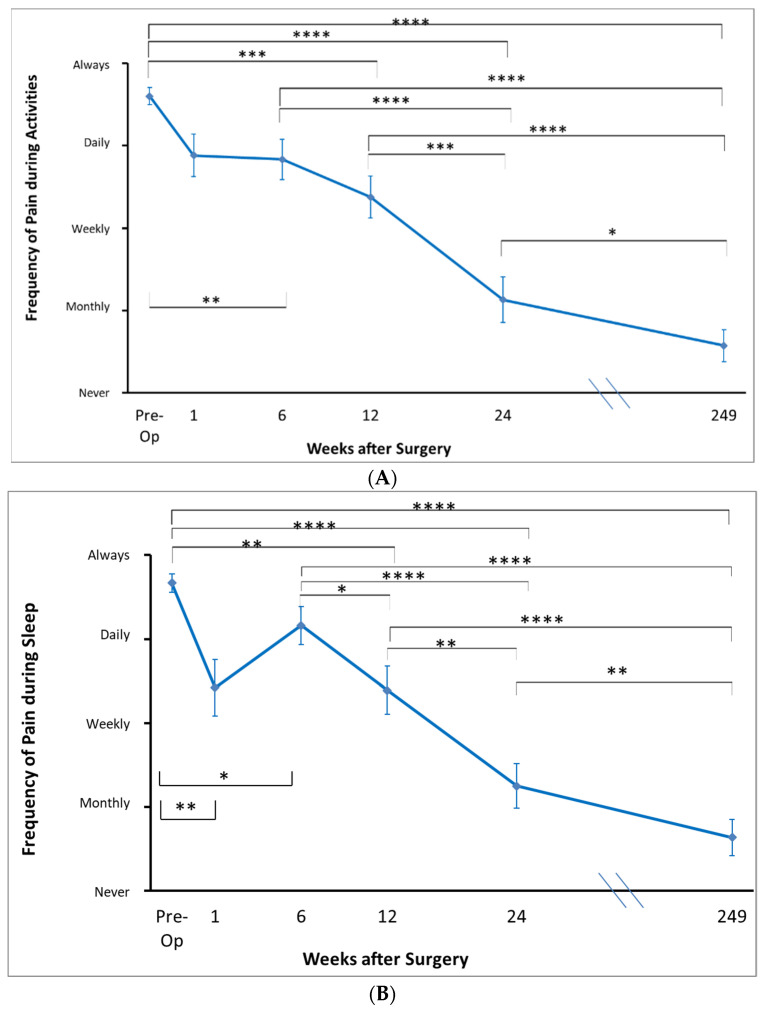

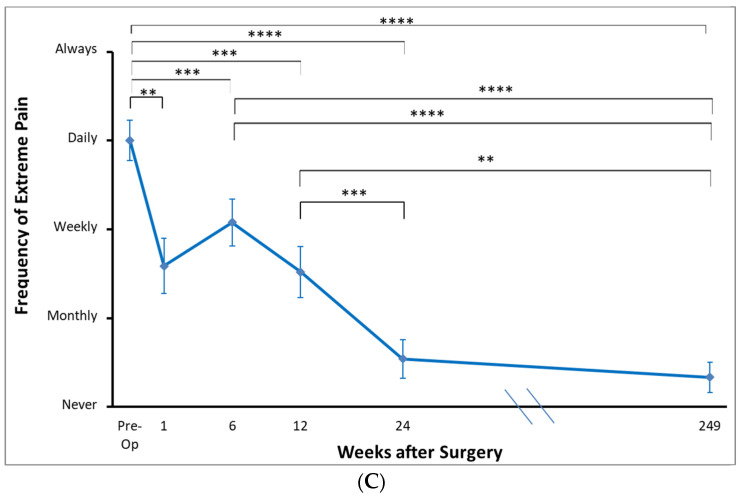
Patient-determined frequency of pain, including during activities (**A**), sleep (**B**), and frequency of extreme pain (**C**). * *p* < 0.05. ** *p* < 0.01. *** *p* < 0.001. **** *p* < 0.0001.

With regards to pre-operative pain frequency, patients reported their frequency of pain with overhead activities and pain during sleep between ‘daily’ and ‘always’, while the frequency of extreme pain was between ‘weekly’ and ‘always’ (Figure 3).

**Figure 4:**
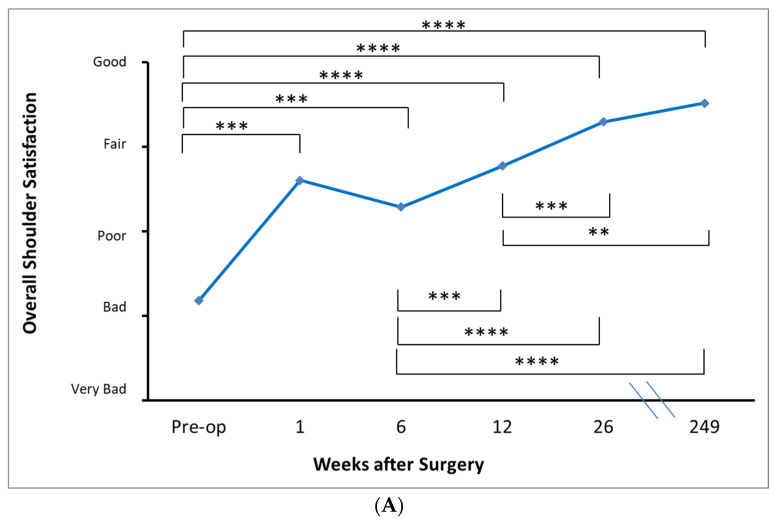

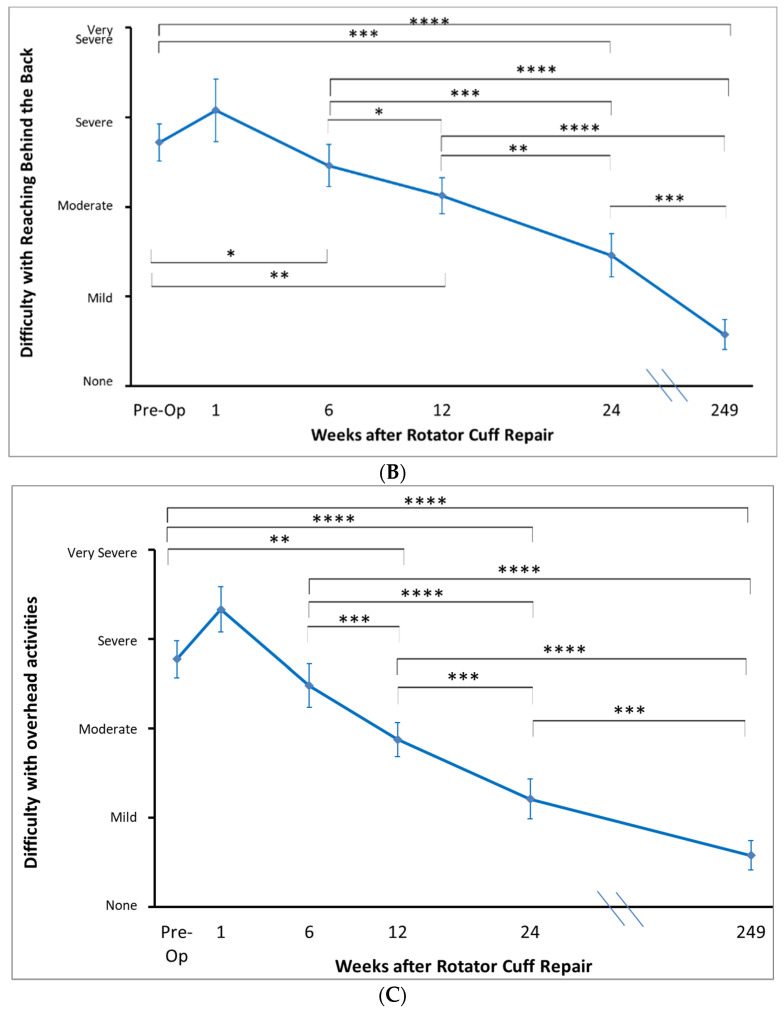
Patient-determined outcomes of overall shoulder function, including patient’s impression of their shoulder overall (**A**), difficulty with reaching behind the back (**B**), and difficulty with overhead activities (**C**). * *p* < 0.05. ** *p* < 0.01. *** *p* < 0.001. **** *p* < 0.0001.

The cohort ranked their overall shoulder status between ‘poor’ and ‘very bad’ (Figure 4). The cohort was also found to have ‘very severe’ difficulty performing overhead activities and activities behind the back.

At the one-week mark following the arthroscopic calcific debridement, on average, patients reported improvements in the level of resting pain from ‘moderate’ to ‘mild’ (*p* = 0.001). The frequency of extreme pain also decreased from ‘daily’ to between ‘none’ and ‘weekly’ (*p* = 0.005). The level of pain during sleep decreased from between ‘severe’ and ‘moderate’ to between ‘moderate’ and ‘mild’ (*p* = 0.004), while the frequency of pain during sleep decreased from ‘always’ to between ‘daily’ and ‘weekly’ (*p* = 0.005) (Figure 2).

These improvements in the reduction of shoulder pain severity and frequency continued over the first six months. From pre-operatively to the 6-month timepoint, there was a significant reduction in the severity of pain at rest (*p* < 0.001), pain with overhead activity (*p* < 0.001) and pain during sleep (*p* < 0.001). There were significant decreases in the frequency of extreme pain (*p* < 0.001), pain with activity (*p* < 0.001), and pain during sleep (*p* < 0.001). By 6 months, patients reported their level of resting pain, the level of pain with overhead activity, and during sleep to be ‘mild’. The frequency of extreme pain was reported as between ‘monthly’ and ‘none’ and the frequency of pain during overhead activity and extreme pain decreased to ‘monthly’.

Patient-rated pain scores at long-term follow up (4.8 years on average) revealed ongoing significant improvements compared to pre-operative values in all components. On average, patients reported their level of resting pain, level of pain with overhead activity, and level of pain during sleep as being between ‘none’ and ‘mild’. At long term follow up, patients reported the frequency of extreme pain as ‘never’ and frequency of pain with overhead activity and pain during sleep as between ‘never’ and ‘monthly’ (Figure 2). Patients rated the state of their shoulder overall as between ‘fair’ and ‘good’. The cohort rated the difficulty performing activities with their arms behind their backs and overhead as between ‘none’ and ‘mild’ (Figure 3).

Between the six-month follow-up and the long-term follow-up, the level and frequency of pain experienced by patients during overhead activity and during sleep were reduced significantly (Figure 2).

### 3.3. Range of Motion and Patient-Reported Stiffness

There were 26 patients (27 shoulders) out of the 31 (33 shoulders) who attended long term follow-up for shoulder assessment and examination of range of motion.

**Figure 5:**
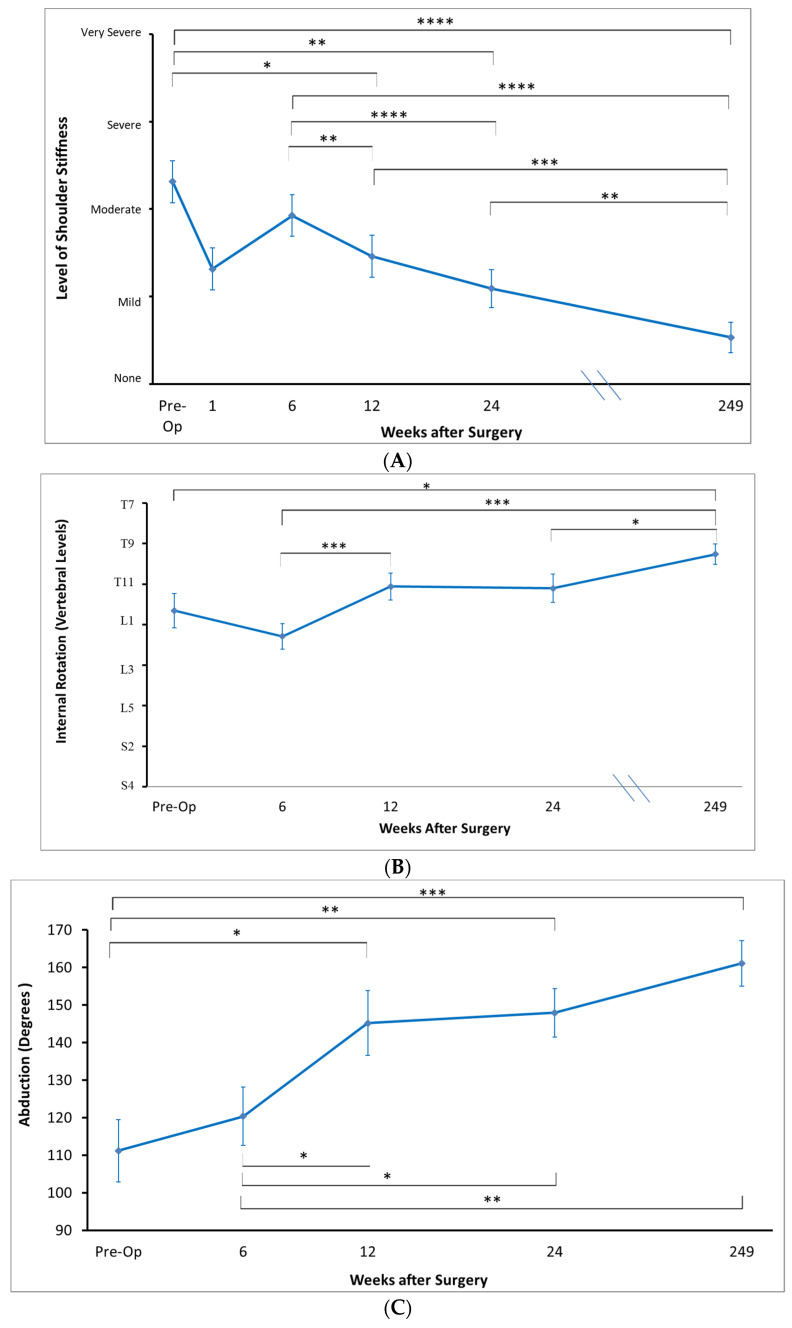
Patient’s reported level of stiffness (**A**), internal rotation range of motion (**B**), and abduction range of motion (**C**). * *p* < 0.05. ** *p* < 0.01. *** *p* < 0.001. **** *p* < 0.0001.

From pre-operative to long-term follow-up (~4.8 years), patients demonstrated statistically significant increases in the range of motion (Figure 5) of abduction (142° ± 8° to 171° ± 4°, *p* = 0.002), forward flexion (111° ± 8° to 161° ± 6°, *p* = 0.003). The range of internal rotation had improved on average from vertebral level T12 (range L1 to T11) to T10 (range T11 to T9) (*p* = 0.013). There was also a trend of improvement in external rotation range of motion over this time period (55° ± 4° to 66° ± 4°, *p* = 0.06).

When comparing between six-month follow-up and long-term follow-up, the abduction range improved significantly from 148° ± 7° to 161° ± 6° (*p* = 0.002). The external rotation had also improved from 48° ± 5° to 66° ± 4° (*p* = 0.03).

With regards to patient-reported stiffness, patients experienced the highest levels of stiffness pre-operatively, and stiffness levels trended downward over time. Pre-operatively, patients reported their stiffness ranging on average from “moderate to quite”. By long-term follow-up, the average stiffness level was “none to a little”, a statistically significant difference (*p* < 0.001). There were statistically significant reductions in patient-reported stiffness from pre-operatively to 6 weeks (*p* = 0.01), pre-operatively to 12 weeks (*p* = 0.01), pre-operatively to 24 weeks (*p* = 0.003), 6 weeks to 12 weeks (*p* = 0.002), and from 6 months to long term follow up (*p* = 0.003).

At long-term follow-up, only one patient reported very severe stiffness; this patient experienced very severe stiffness pre-operatively and throughout all post-operative follow-up appointments. One patient reported their shoulder as “Quite” stiff at long-term follow-up, and this patient had reported his shoulder as “Quite” stiff pre-operatively also. Out of the 27 patients, only one patient (3%) reported their shoulder as stiffer at long-term follow-up compared to pre-operatively; this patient’s stiffness level had increased from “a little” stiff to “moderately” stiff.

### 3.4. Strength

A total of 26 patients (27 shoulders) out of 31 patients (33 shoulders) completed the strength-testing follow-up.

**Figure 6:**
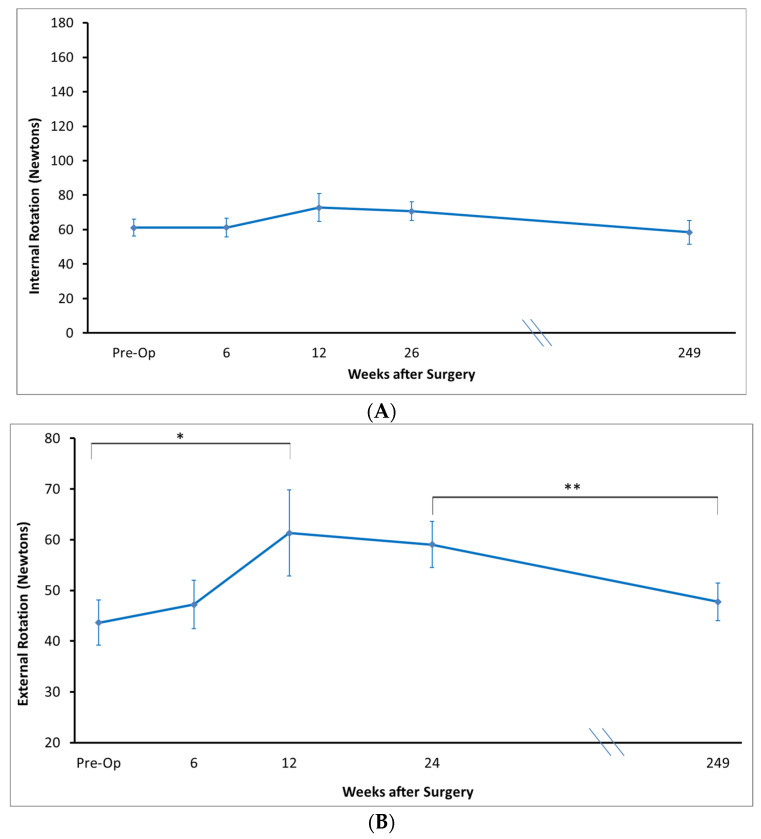

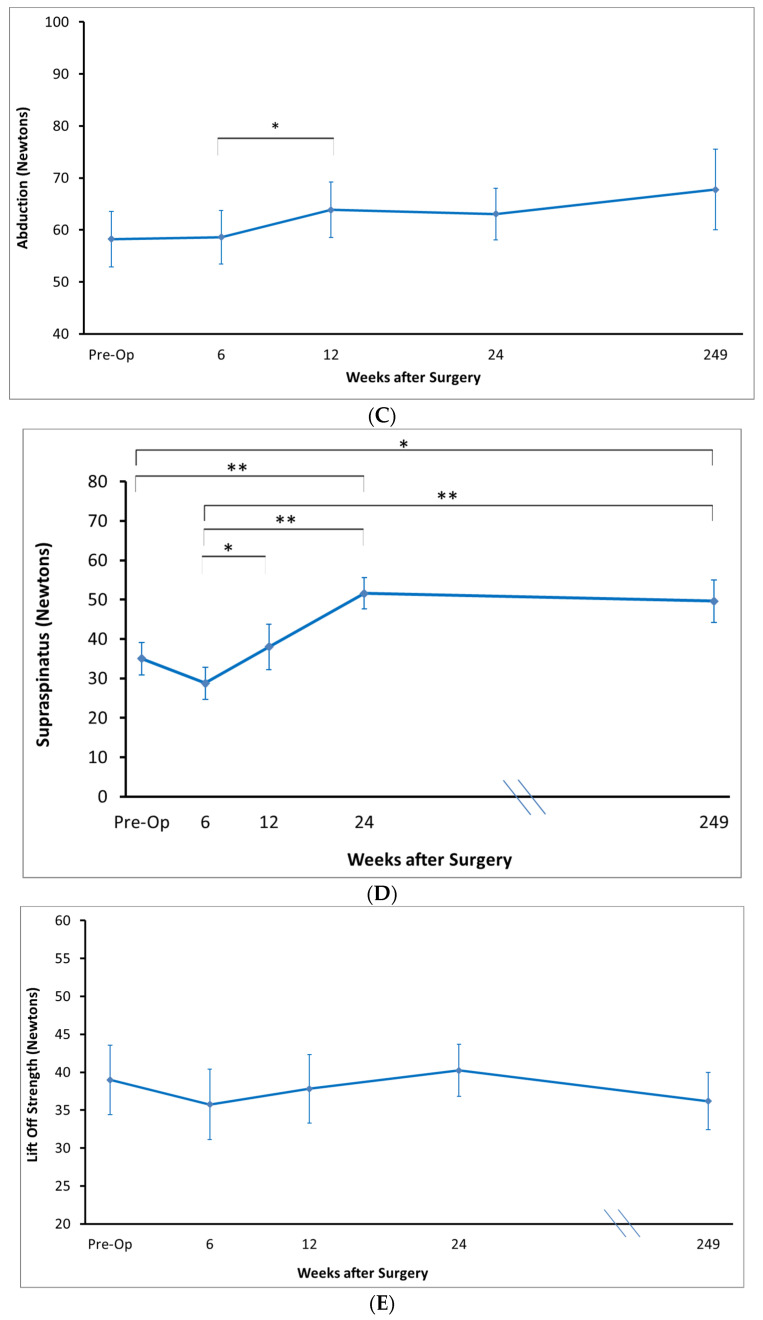
Shoulder strength in (**A**) internal rotation (**B**) external rotation, (**C**) abduction (**D**) supraspinatus testing (**E**) Lift off. * *p* < 0.05. ** *p* < 0.01.

There was improvement in strength (Figure 6) on testing the supraspinatus from preoperatively (35 ± 4 N) to 6 months (52 ± 4 N) (*p* = 0.005), and from preoperatively to long term follow-up (50 ± 5 N) (*p* = 0.02), but no difference from 6 months to long-term follow-up (*p* = 0.98).

With regards to abduction, internal rotation, and lift-off, there were no significant differences in strength from pre-operatively to post-operatively at all timepoints.

External rotation strength improved from pre-operatively to 12 weeks (44 ± 5 N to 61 ± 8 N, *p* = 0.04), however, there was no significant difference between pre-operative and long-term external rotation strength (44 ± 5 N to 47 ± 4 N, *p* = 0.92). There was no significant difference between strength at 12 weeks and strength at long-term follow-up (61 ± 4 N to 47 ± 4 N, *p* = 0.22).

### 3.5. Imaging/Radiographic Findings

Regarding X-ray and ultrasound imaging (27 patients), 24 out of 27 (89%) re-imaged shoulders had no residual calcifications at long-term follow-up. There were one (4%) medium-sized (5–15 mm) and two (7%) small-sized (<5 mm) residual calcium deposits that were identified. A total of 26 shoulders out of 27 (96%) images had intact rotator cuffs at a mean of 4.8 years of follow-up. One shoulder out of 27 (4%) imaged had a 10 mm × 10 mm 90% undersurface tear of the supraspinatus at long-term follow-up that required secondary ipsilateral surgery.

### 3.6. Complications

There were no intra-operative complications in the 31 patients (33 shoulders). One male patient experienced a wound infection at one-week post-operative and was treated with arthroscopy of the sub-acromial space, wound incision, and debridement in addition to antibiotics. There was no development of post-operative osteoarthritis or shoulder instability in the ipsilateral shoulder noted during the study period.

## 4. Discussion

This study validated the efficacy of arthroscopic debridement of calcific tendonitis with localization assistance from a breast biopsy needle under ultrasound guidance. Patients experienced significant improvements within the first week post-operatively with regards to shoulder function and pain and continued to make further improvements until a mean post-operative time of 4.8 years.

Our results provided further evidence of the short-term effectiveness of this procedure. By only one week post-surgery, patients reported significant reductions in pain severity at rest and during sleep, as well as reduced frequency of pain during sleep and reduced frequency of extreme pain. By 6 months post-operatively, patients in our study, on average, reported “mild” shoulder pain on a “monthly” basis, whereas pre-operatively, patients experienced pain either daily or always, and of moderate to severe intensity. These findings of the short-term effectiveness of this technique were consistent with those of Kelly et al. [29], who also showed a reduction of pain frequency and severity by 6 months post-surgery.

By long-term follow-up (mean 4.8 years), patients demonstrated dramatic improvements in all of the study’s measurements of pain severity and frequency, function, overall satisfaction, and stiffness compared to pre-operatively. Furthermore, they also experienced improvements in almost all measures of range of motion compared to pre-operatively, except for external rotation, which was un-changed. Notably, patients continued to show further improvements between the 6-month mark and the long-term follow-up with regards to range of motion (in internal rotation and forward flexion), subjective stiffness, pain frequency (during sleep and during activities), and pain severity (during sleep and during overhead activities).

This technique was associated with the preservation of the long-term structural integrity of the rotator cuff. Within the study group, 22 patients out of 33 also had concurrent rotator cuff repairs. However, by long-term follow-up, only 1 out of 27 (4%) shoulders suffered a torn rotator cuff. This figure compares favorably to arthroscopic rotator cuff repair, which has retear rates documented between 11% and 94% [20,26].

Yoo et al. [30] performed a similar study on 35 patients who underwent arthroscopic treatment for calcific tendonitis, with a median follow-up of 2.5 years. Their operative technique also aimed for complete removal of calcific material; however, it differed from our technique as they relied on visual identification of calcifications via arthroscopy without the use of ultrasound or a breast biopsy needle. For calcifications that were difficult to visualize, they occasionally used percutaneous spinal needle insertion without ultrasound guidance. Their technique resulted in complete calcium removal in 29/35 patients (86%) compared to 24/27 (89%) in our cohort. Their cohort demonstrated improvement in pain relief and functionality similar to our study, with a visual analogue score for pain improving from 6 ± 2 to 1 ± 1 at final follow-up (*p* < 0.001) and a constant score improving from 60 (±20) preoperatively to 87 (±15) at final follow-up (*p* < 0.001). However, 10 patients out of the 35 patients (29%) who did not experience any preoperative limitation to motion, developed a secondary stiff shoulder, which ultimately resolved by 3–9 months post-operatively for all patients. Their rehabilitation protocol was “similar to that of a small rotator cuff tear”, and our facility also used our rotator cuff rehabilitation protocol when a repair was performed. Our technique, utilizing a breast biopsy needle and ultrasound guidance, resulted in minimal post-operative stiffness and significant improvements in range of motion. By 6 weeks, only one patient (1/27, 4%) that did not have preoperative stiffness had developed a secondary stiff shoulder. On average, stiffness improved from “moderate to quite” pre-operatively, to “mild” at 6-month follow-up. At long-term follow-up, out of the 32 patients, only one patient (3%) reported their shoulder as stiffer at long-term follow-up compared to pre-operatively. Additionally, only one patient reported very severe stiffness (this patient had experienced very severe stiffness since pre-operatively), and one patient reported their shoulder to be “Quite” stiff (this patient had reported his shoulder as “Quite” stiff pre-operatively also). These results suggest that accurate localization of the calcium deposit with the use of the breast biopsy needle under ultrasound guidance may result in reduced stiffness post-operatively. The minimal stiffness encountered post-operatively by this technique may be explained by a more thorough removal of calcium, as secondary stiffness post-arthroscopic debridement has been thought to be due to residual calcium fragments inducing further inflammation within the surrounding structures [17,20].

The substantial pain relief that this procedure provides to patients may be explained by enhanced accuracy in the removal of the calcific deposit. By long-term follow-up, none of the shoulders had any large calcium deposits remaining, only one patient had a medium-sized deposit, and two still had small calcium deposits (<5 mm) remaining. Hackett et al. [4] demonstrated that calcium deposits were associated with a significant increase in neo-vascularization, neo-innervation, macrophages, and mast cells compared to rotator cuff tears alone, and postulated that the calcific material was responsible for inciting inflammation and causing pain. Our study’s findings are consistent with this hypothesis, as accurate debridement of the calcific material utilizing localization assistance from a breast needle biopsy resulted in clinically significant improvements in patients’ pain severity during sleep and at rest quite rapidly by as early as 1 week post-operatively, with further gains to be attained over time.

The strengths of our study are the holistic long-term follow-up comparisons of patient-rated and examiner-rated data, radiological outcomes, and assessment via ultrasound of rotator cuff integrity. The internal validity of the study is high, with a single orthopedic surgeon and sonographer performing the surgery and follow-up assessments, respectively. This may reduce the external validity of this study. Other weaknesses include the lack of a control group. However, to find a similar group without surgical treatment with similar follow-up intervals is difficult, and administration of a placebo would, in our view, have ethical issues. The findings are only relevant to those with symptoms sufficiently severe to require/elect surgery.

## 5. Conclusions

This study shows that:

○ Arthroscopic debridement of calcific tendonitis with localization assistance from a breast biopsy needle under ultrasound guidance is successful in removing the rotator cuff calcific deposit and maintaining the integrity the of rotator cuff tendons.
○ At a mean of 4.8 years, arthroscopic debridement of calcific tendonitis was successful in relieving the severity and frequency of pain with overhead activities, pain at rest, and pain during sleep.
○ Patients experienced significant improvements in range of motion.
○ Secondary shoulder stiffness was not a significant complication of this technique.

## Figures and Tables

**Table 1:** Demographics of the study population.

Item	Data
Sex	10 males (32%) 21 females (68%)
Side	18 Right shoulders (54%) 15 Left Shoulders (46%)
Age	Mean: 54 years Range: 38–76 years
Time of Long-Term Follow-Up	Mean: 4.8 years Range: 2–10 years
Concurrent Rotator Cuff Repair performed?	22 shoulders had concurrent rotator cuff repairs 11 shoulders did not have concurrent rotator cuff repairs

## Data Availability

The data will be made available in a publicly accessible repository (Supplementary Materials).

## Supplementary Materials

The following supporting information can be downloaded at: https://www.mdpi.com/article/10.3390/jcm12093114/s1.

## References

[1] Elshewy M.T. (2016). Calcific tendinitis of the rotator cuff. World J. Orthop..

[2] Ellman H. (1987). Arthroscopic subacromial decompression: Analysis of one- to three-year results. Arthrosc. J. Arthrosc. Relat. Surg..

[3] Albert J.D., Meadeb J., Guggenbuhl P. (2007). High-energy extracorporeal shock-wave therapy for calcifying tendinitis of the rotator cuff: A randomised trial. J. Bone Jt. Surg. Br..

[4] Hackett L., Millar N.L., Lam P., Murrell G.A. (2016). Are the Symptoms of Calcific Tendinitis Due to Neoinnervation and/or Neovascu-larization?. J. Bone Jt. Surg. Am..

[5] Bannuru R.R., Flavin N.E., Vaysbrot E., Harvey W., McAlindon T. (2014). High-energy extracorporeal shock-wave therapy for treating chronic calcific tendinitis of the shoulder: A systematic review. Ann. Intern. Med..

[6] Cadogan A., Laslett M., Hing W., McNair P., Williams M. (2011). Reliability of a new hand-held dynamometer in measuring shoulder range of motion and strength. Man. Ther..

[7] Carlisi E., Lisi C., Dall’Angelo A., Monteleone S., Nola V., Tinelli C., Toffola E.D. (2018). Focused extracorporeal shock wave therapy combined with supervised eccentric training for supraspinatus calcific tendinopathy. Eur. J. Phys. Rehabil. Med..

[8] Daecke W., Kusnierczak D., Loew M. (2002). Long-term effects of extracorporeal shockwave therapy in chronic calcific tendinitis of the shoulder. J. Shoulder Elb. Surg..

[9] Del Castillo F., Ramos Álvarez J.J., Rodriguez Fabián G., González Pérez J., Jiménez Herranz E., Varela E. (2016). Extracorporeal shockwaves versus ultrasound-guided percutaneous lavage for the treatment of rotator cuff calcific tendinopathy: A randomised controlled trial. Eur. J. Phys. Rehabil. Med..

[10] Farr S., Sevelda F., Mader P., Graf A., Petje G., Sabeti-Aschraf M. (2011). Extracorporeal shockwave therapy in calcifying tendinitis of the shoulder. Knee Surg. Sports Traumatol. Arthrosc..

[11] Hayes K., Walton J.R., Szomor Z.L., Murrell G.A. (2002). Reliability of 3 methods for assessing shoulder strength. J. Shoulder Elb. Surg..

[12] Hayes K., Walton J.R., Szomor Z.L., Murrell G.A. (2001). Reliability of five methods for assessing shoulder range of motion. Aust. J. Physiother..

[13] Hsu C.J., Wang D.Y., Tseng K.F., Fong Y.C., Hsu H.C., Jim Y.F. (2008). Extracorporeal shock wave therapy for calcifying tendinitis of the shoulder. J. Shoulder Elb. Surg..

[14] Arirachakaran A., Boonard M., Yamaphai S., Prommahachai A., Kesprayura S., Kongtharvonskul J. (2016). Extracorporeal shock wave therapy, ultrasound-guided percutaneous lavage, corticosteroid injection and combined treatment for the treatment of rotator cuff calcific tendinopathy: A network meta-analysis of RCTs. Eur. J. Orthop. Surg. Traumatol..

[15] Ioppolo F., Tattoli M., Di Sante L., Attanasi C., Venditto T., Servidio M., Cacchio A., Santilli V. (2012). Extracorporeal Shock-Wave Therapy for Supraspinatus Calcifying Tendinitis: A Randomized Clinical Trial Comparing Two Different Energy Levels. Phys. Ther..

[16] Jerosch J., Strauss J., Schmiel S. (1998). Arthroscopic treatment of calcific tendinitis of the shoulder. J. Shoulder Elb. Surg..

[17] Kempf J., Bonnomet F., Nerisson D., Gastaud F., Lacaze F., Geraud H. (1997). Arthroscopic Isolated Excision of Rotator Cuff Calcium Deposits.

[18] Lam F., Bhatia D., van Rooyen K., de Beer J. (2006). Modern management of calcifying tendinitis of the shoulder. Curr. Orthop..

[19] L’Insalata J.C., Warren R.F., Cohen S.B., Altchek D.W., Peterson M.G. (1997). A Self-Administered Questionnaire for Assessment of Symptoms and Function of the Shoulder. J. Bone Jt. Surg..

[20] McColl A.H., Lam P.H., Murrell G.A. (2019). Are we getting any better? A study on repair integrity in 1600 consecutive arthroscopic rotator cuff repairs. JSES Open Access.

[21] Diehl P., Schauwecker J. (2014). Schmerzhafte Schulter—Ist es Kalk?. MMW-Fortschr. Med..

[22] VuMedi Ultrasound-Guided Placement of a Localization Wire for Arthroscopic Treatment of Calcific Tendonitis. https://www.vumedi.com/video/ultrasound-guided-placement-of-a-localization-wire-for-arthroscopic-treatment-of-calcific-tendonitis/.

[23] Murrell G.A.C. (2012). Advances in Rotator Cuff Repair—Undersurface Repair. Tech. Shoulder Elb. Surg..

[24] Molé D., Kempf J.F., Gleyze P., Rio B., Bonnomet F., Walch G. (1993). Results of endoscopic treatment of non-broken tendinopathies of the rotator cuff. 2. Calcifications of the rotator cuff. Rev. Chir. Orthop. Reparatrice Appar. Mot..

[25] Osbahr D.C., Murrell G.A.C. (2006). The Rotator Cuff Functional Index. Am. J. Sports Med..

[26] Plachel F., Jo O.I., Rüttershoff K., Andronic O., Ernstbrunner L. (2022). A Systematic Review of Long-term Clinical and Radiological Outcomes of Arthroscopic and Open/Mini-open Rotator Cuff Repairs. Am. J. Sports Med..

[27] Ricci V., Mezian K., Chang K.-V., Özçakar L. (2022). Clinical/Sonographic Assessment and Management of Calcific Tendinopathy of the Shoulder: A Narrative Review. Diagnostics.

[28] Uhthoff H.K., Loehr J.W. (1997). Calcific Tendinopathy of the Rotator Cuff: Pathogenesis, Diagnosis, and Management. J. Am. Acad. Orthop. Surg..

[29] Kelly M.J., Andres B., Briggs L., Lam P., Ali R., Murrell G.A. (2012). Ultrasound-guided Placement of a Localization Wire for Arthroscopic Treatment of Calcific Tendonitis. Tech. Shoulder Elb. Surg..

[30] Yoo J.C., Park W.H., Koh K.H., Kim S.M. (2010). Arthroscopic treatment of chronic calcific tendinitis with complete removal and rotator cuff tendon repair. Knee Surg. Sport. Traumatol. Arthrosc..

