# Supplementary material for: Ultrasound Guided Arthroscopic Removal of Calcific Tendonitis: A Minimum of 2-Year Followup
Source: J Clin Med. 2023 Apr 25;12(9):3114. doi: 10.3390/jcm12093114 (PMC10179588; doi:10.3390/jcm12093114)
Supplement: Supplementary file 1 [file jcm-12-03114-s001.zip › Generic Graph Templates/Difficulty with overhead activities.pptx]

## Slide 1
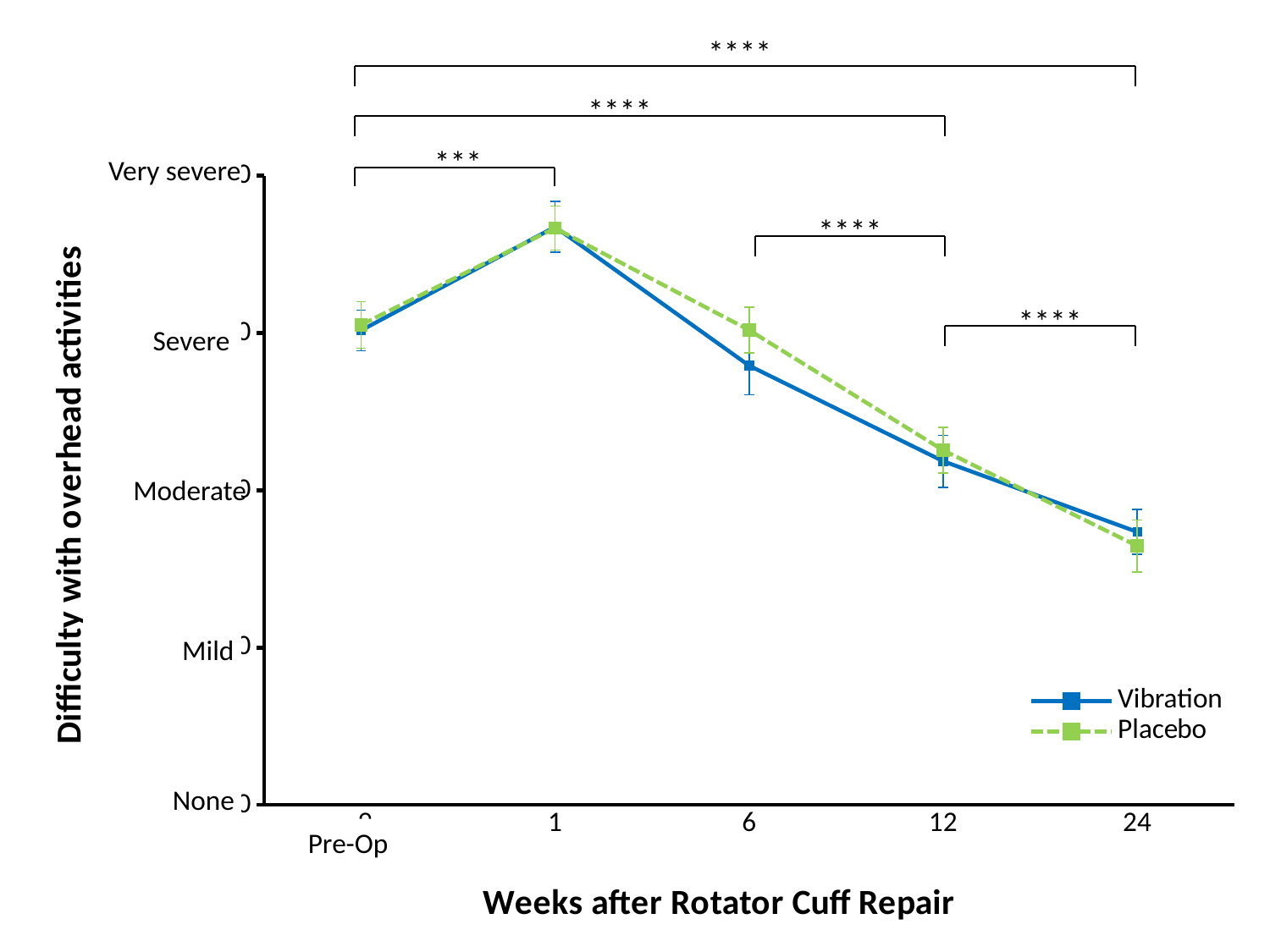

****
****
Very severe
Severe
Moderate
Mild
None
***
### Chart
| Category | Vibration | Placebo |
|---|---|---|
| -9 | 3.016393442622954 | 3.051724137931035 |
| 1 | 3.6744186046511627 | 3.6666666666666665 |
| 6 | 2.7924528301886755 | 3.0181818181818207 |
| 12 | 2.1851851851851847 | 2.2549019607843173 |
| 24 | 1.735849056603774 | 1.6470588235294121 |****
****
Pre-Op

## Slide 2
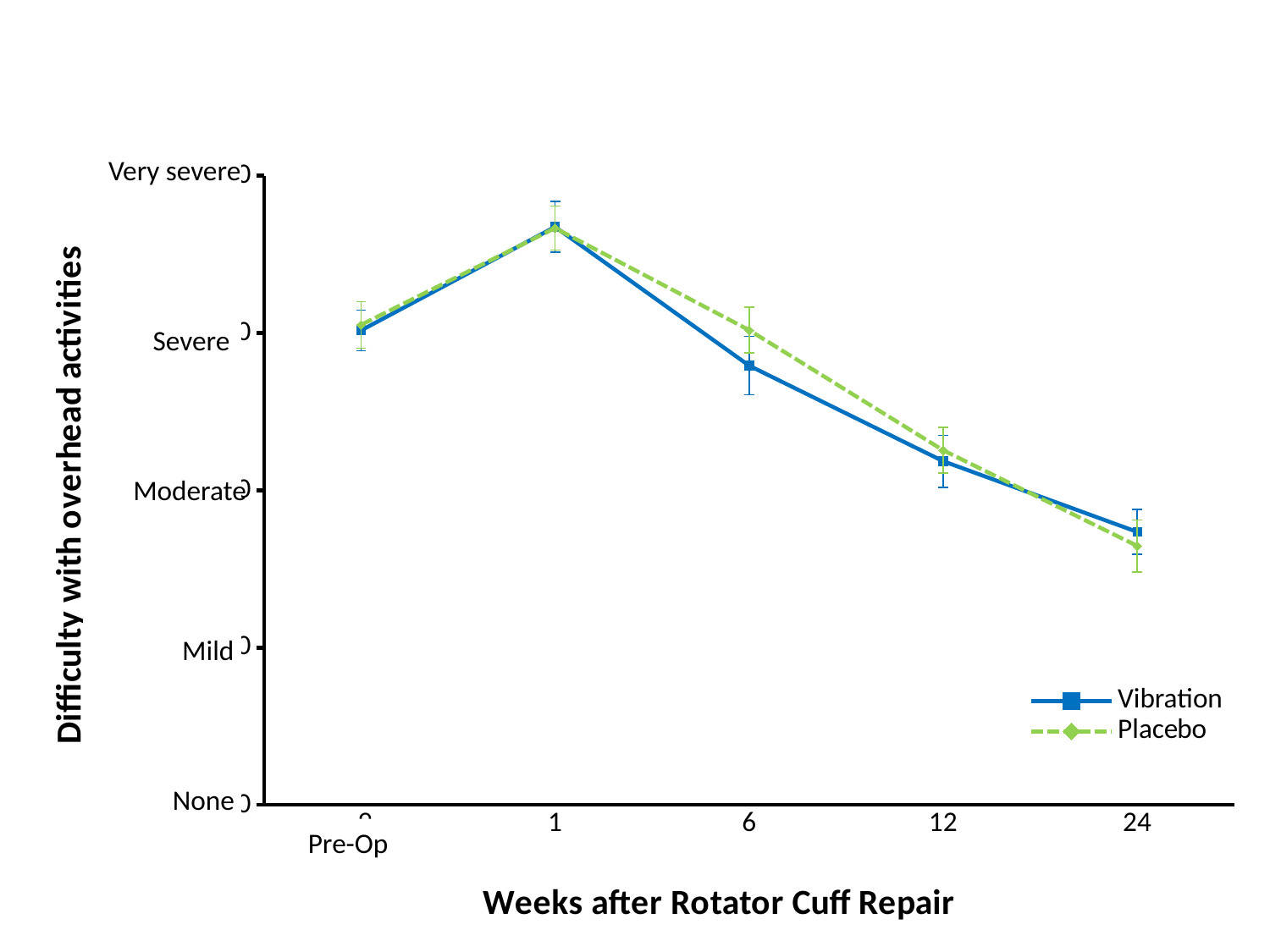

Very severe
Severe
Moderate
Mild
None
### Chart
| Category | Vibration | Placebo |
|---|---|---|
| -9 | 3.016393442622955 | 3.051724137931035 |
| 1 | 3.6744186046511627 | 3.6666666666666665 |
| 6 | 2.7924528301886737 | 3.018181818181821 |
| 12 | 2.1851851851851847 | 2.2549019607843186 |
| 24 | 1.7358490566037739 | 1.6470588235294121 |Pre-Op

## Slide 3
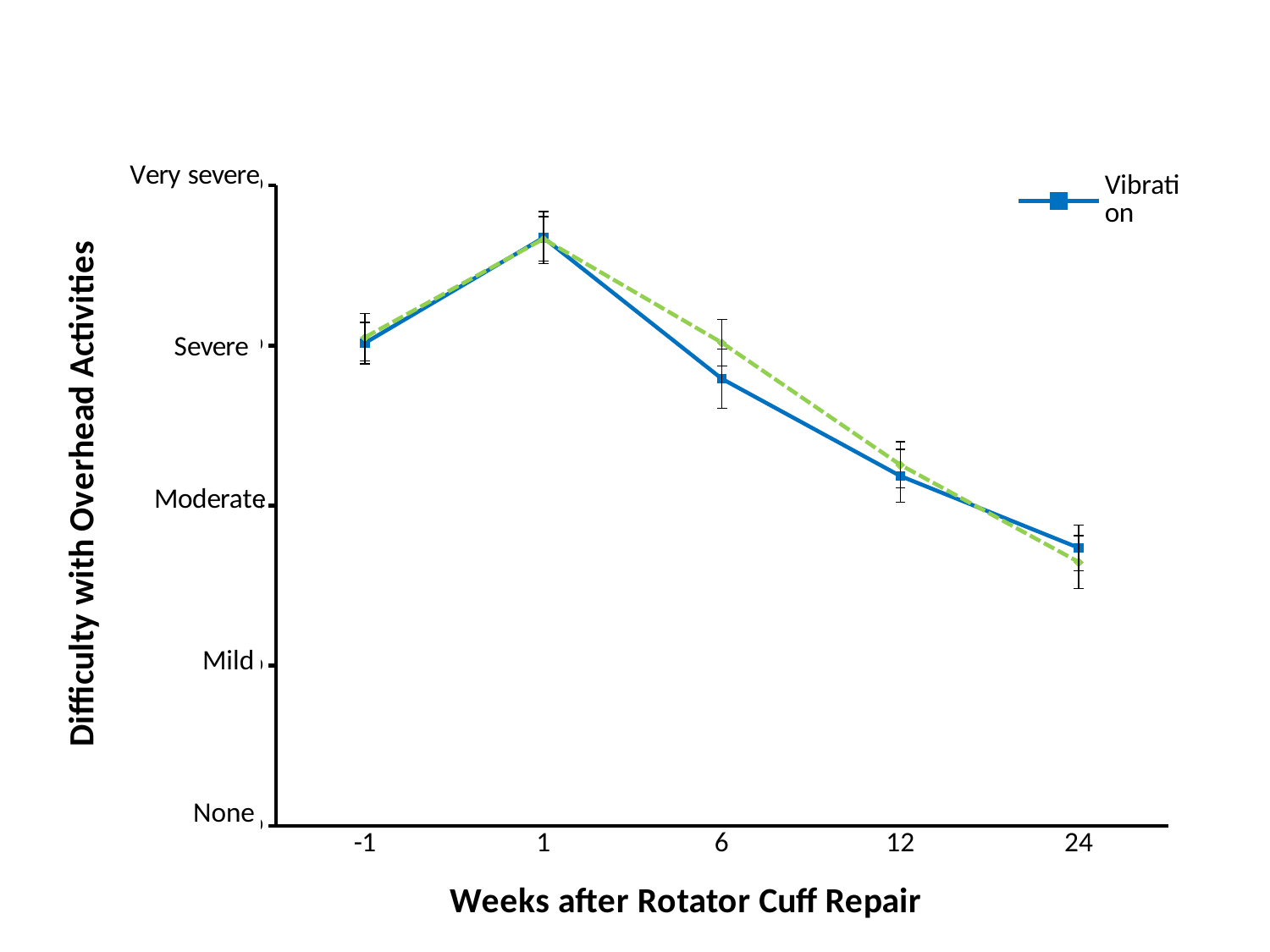

### Chart
| Category | Vibration | Placebo |
|---|---|---|
| -1 | 3.0163934426229555 | 3.051724137931035 |
| 1 | 3.6744186046511627 | 3.6666666666666665 |
| 6 | 2.7924528301886733 | 3.018181818181821 |
| 12 | 2.1851851851851847 | 2.254901960784319 |
| 24 | 1.7358490566037739 | 1.6470588235294121 |

## Slide 4
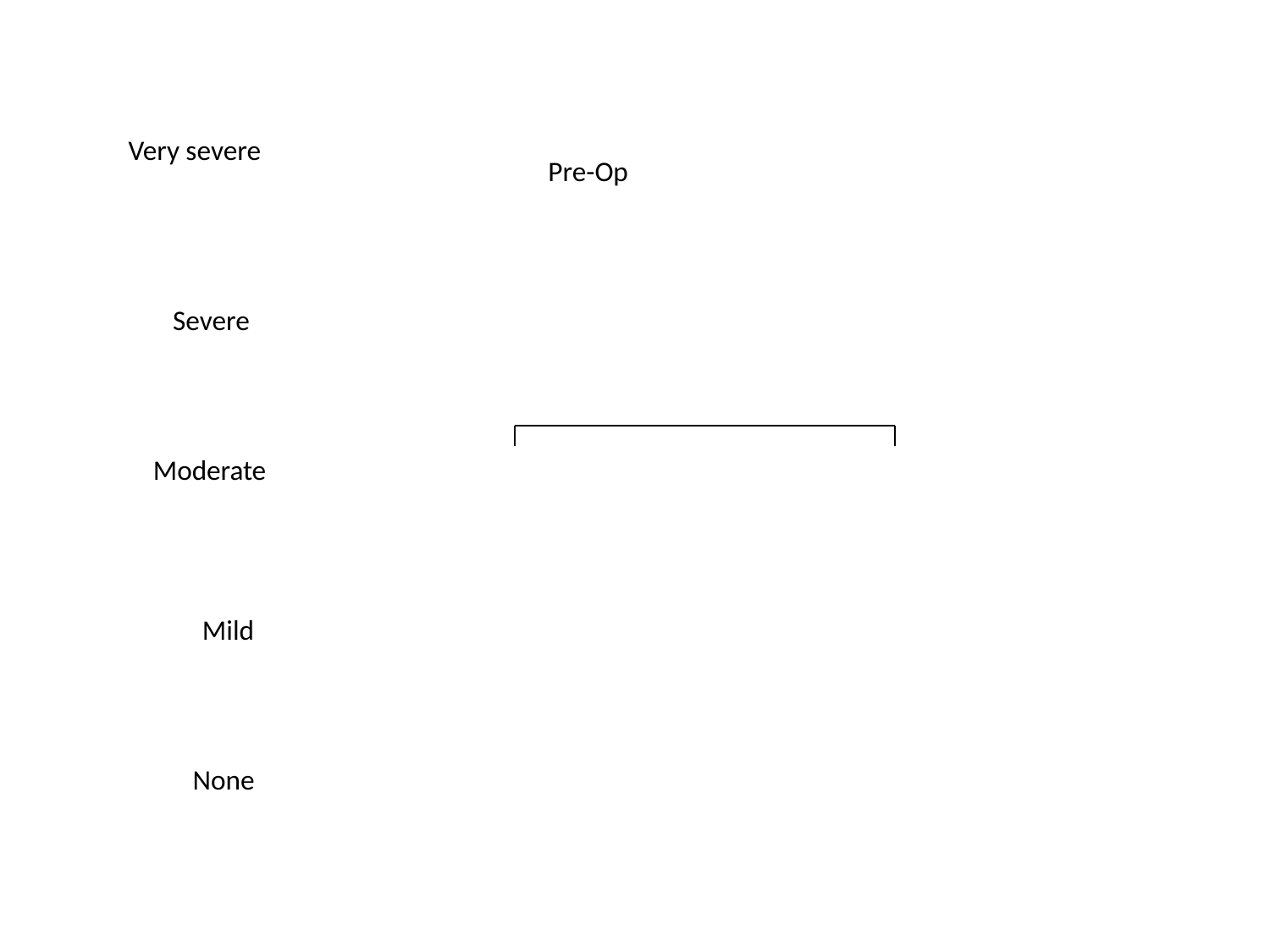

Very severe
Severe
Moderate
Mild
None
Pre-Op
