# Supplementary material for: Ultrasound Guided Arthroscopic Removal of Calcific Tendonitis: A Minimum of 2-Year Followup
Source: J Clin Med. 2023 Apr 25;12(9):3114. doi: 10.3390/jcm12093114 (PMC10179588; doi:10.3390/jcm12093114)
Supplement: Supplementary file 1 [file jcm-12-03114-s001.zip › Generic Graph Templates/Frequency of Pain during Activities.pptx]

## Slide 1
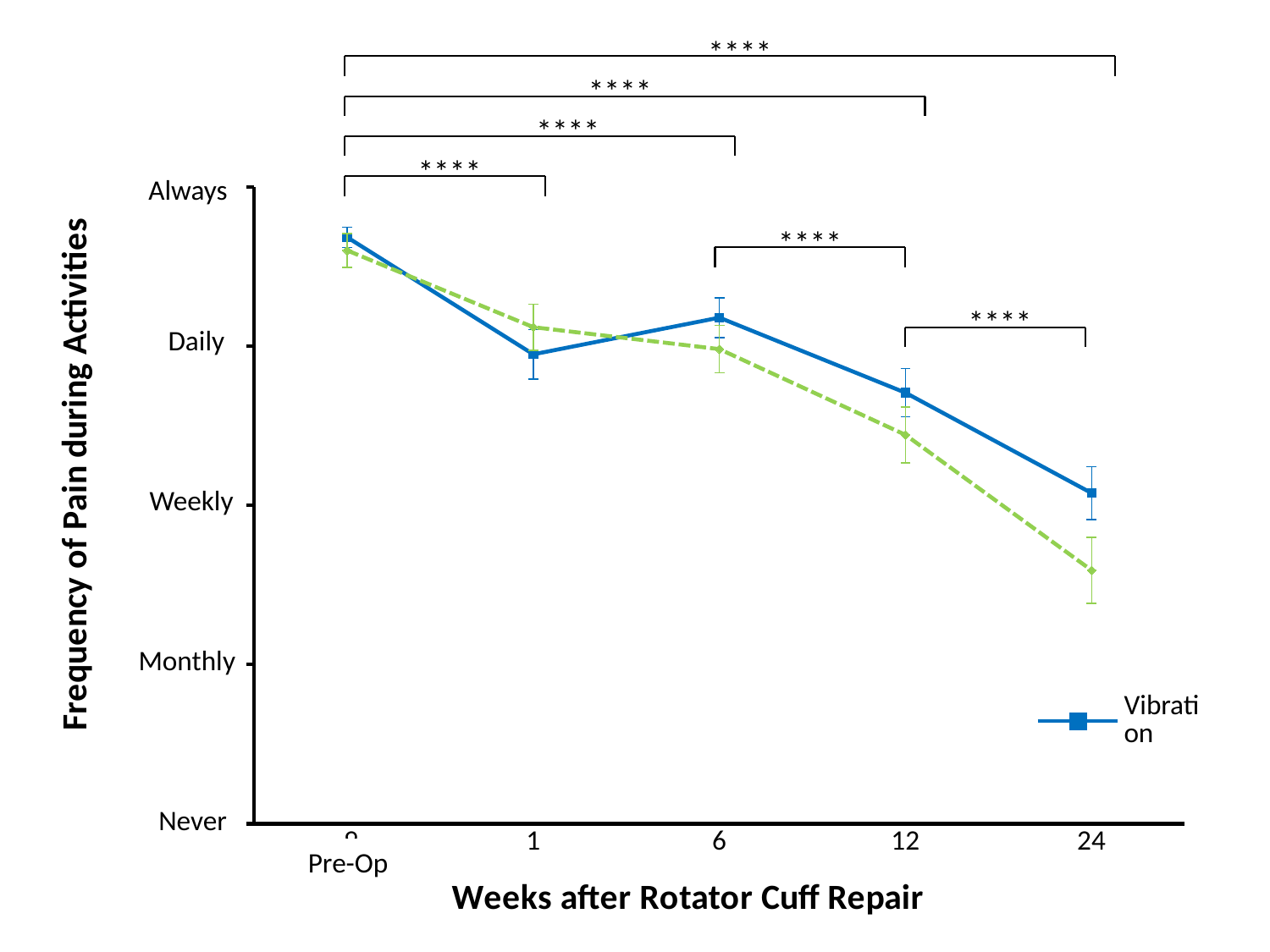

****
****
****
Always
Daily
Weekly
Monthly
Never
****
### Chart
| Category | Vibration | Placebo |
|---|---|---|
| -9 | 3.682539682539683 | 3.6 |
| 1 | 2.9482758620689653 | 3.1186440677966107 |
| 6 | 3.178571428571429 | 2.980769230769231 |
| 12 | 2.7068965517241383 | 2.442307692307693 |
| 24 | 2.076923076923077 | 1.5918367346938775 |****
****
Pre-Op

## Slide 2
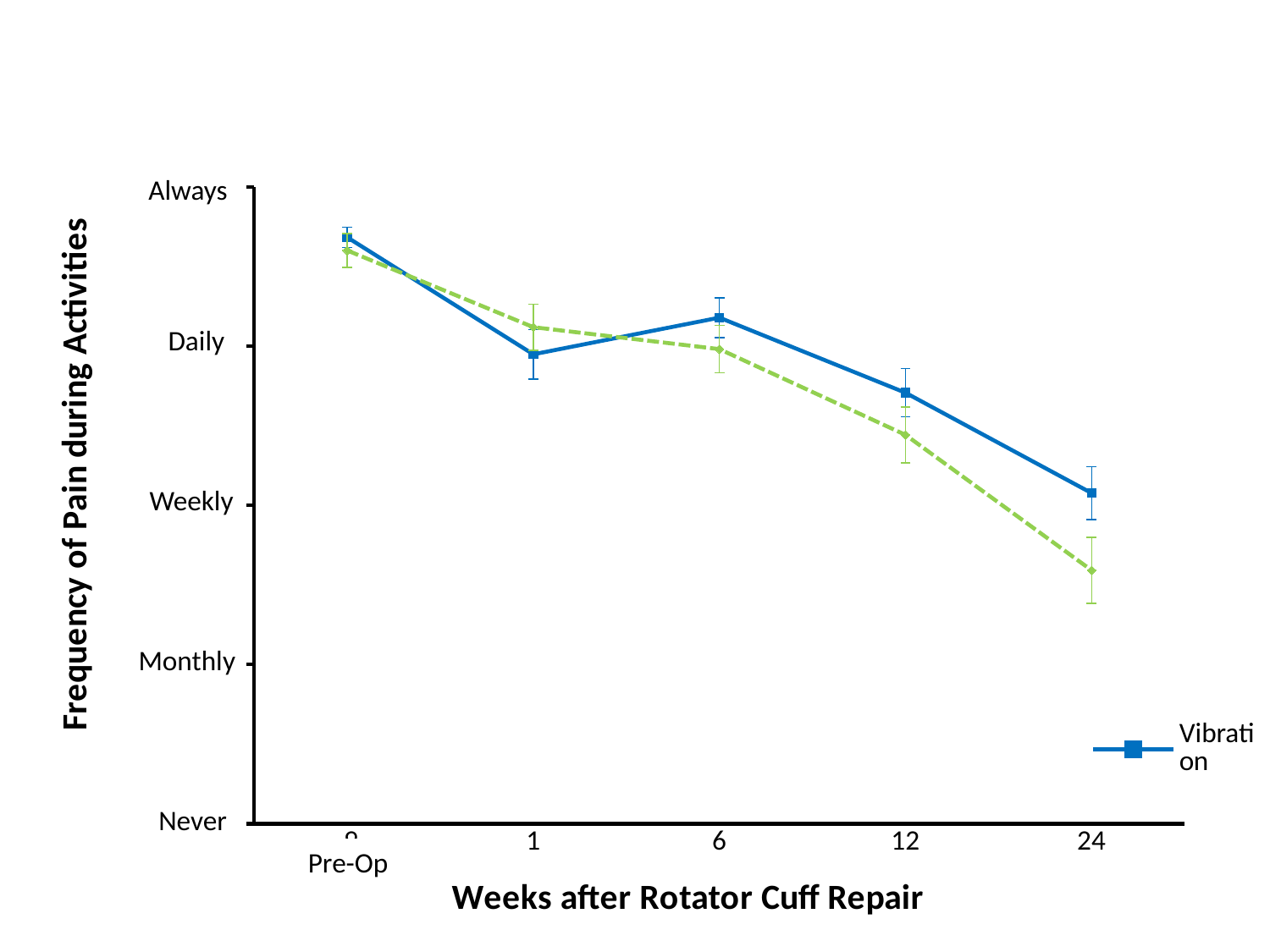

Always
Daily
Weekly
Monthly
Never
### Chart
| Category | Vibration | Placebo |
|---|---|---|
| -9 | 3.6825396825396832 | 3.6 |
| 1 | 2.9482758620689653 | 3.118644067796611 |
| 6 | 3.17857142857143 | 2.980769230769231 |
| 12 | 2.7068965517241392 | 2.4423076923076943 |
| 24 | 2.076923076923077 | 1.5918367346938775 |Pre-Op

## Slide 3
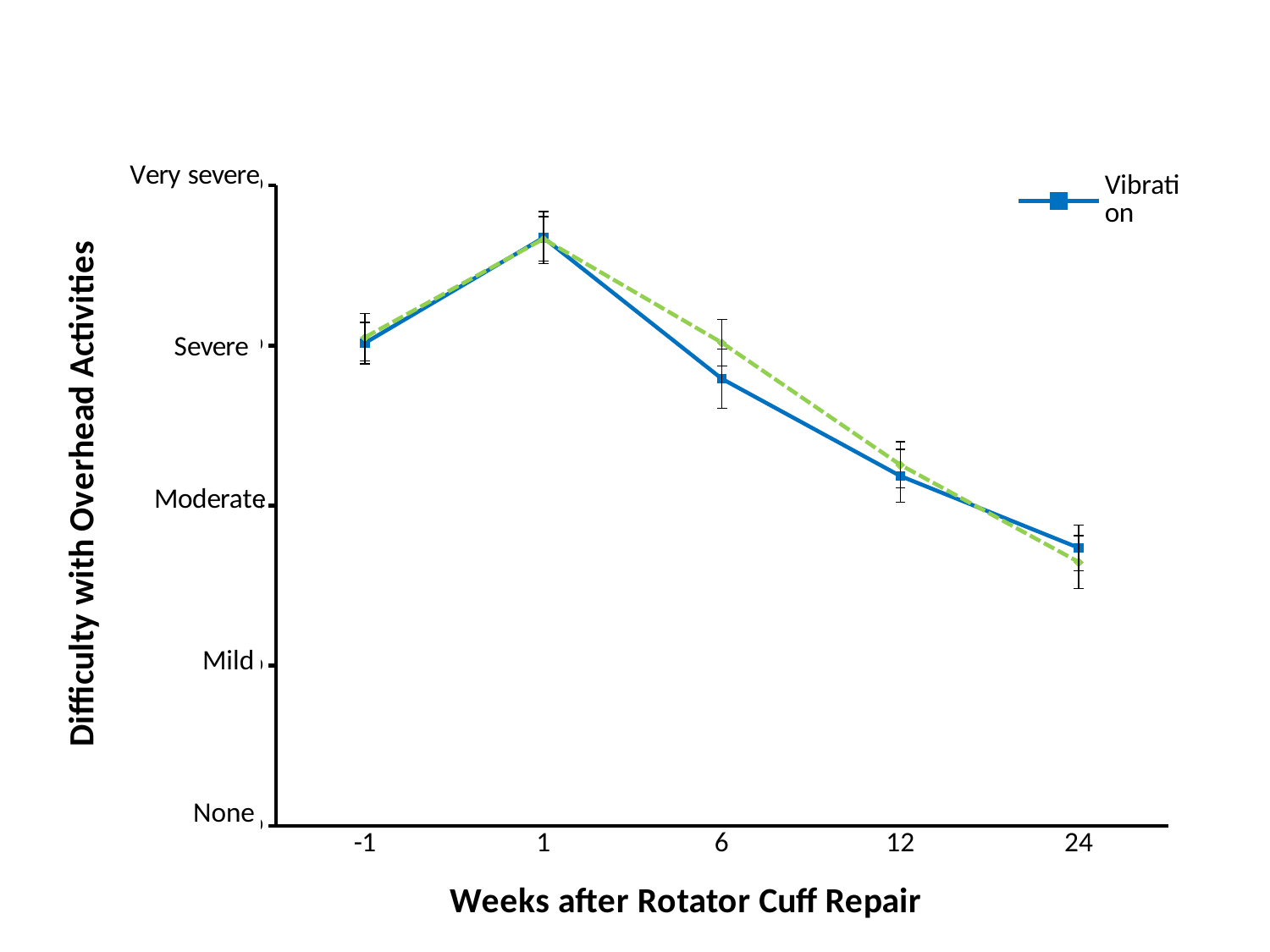

### Chart
| Category | Vibration | Placebo |
|---|---|---|
| -1 | 3.0163934426229546 | 3.051724137931035 |
| 1 | 3.6744186046511627 | 3.6666666666666665 |
| 6 | 2.7924528301886746 | 3.018181818181821 |
| 12 | 2.1851851851851847 | 2.254901960784318 |
| 24 | 1.7358490566037739 | 1.6470588235294121 |

## Slide 4
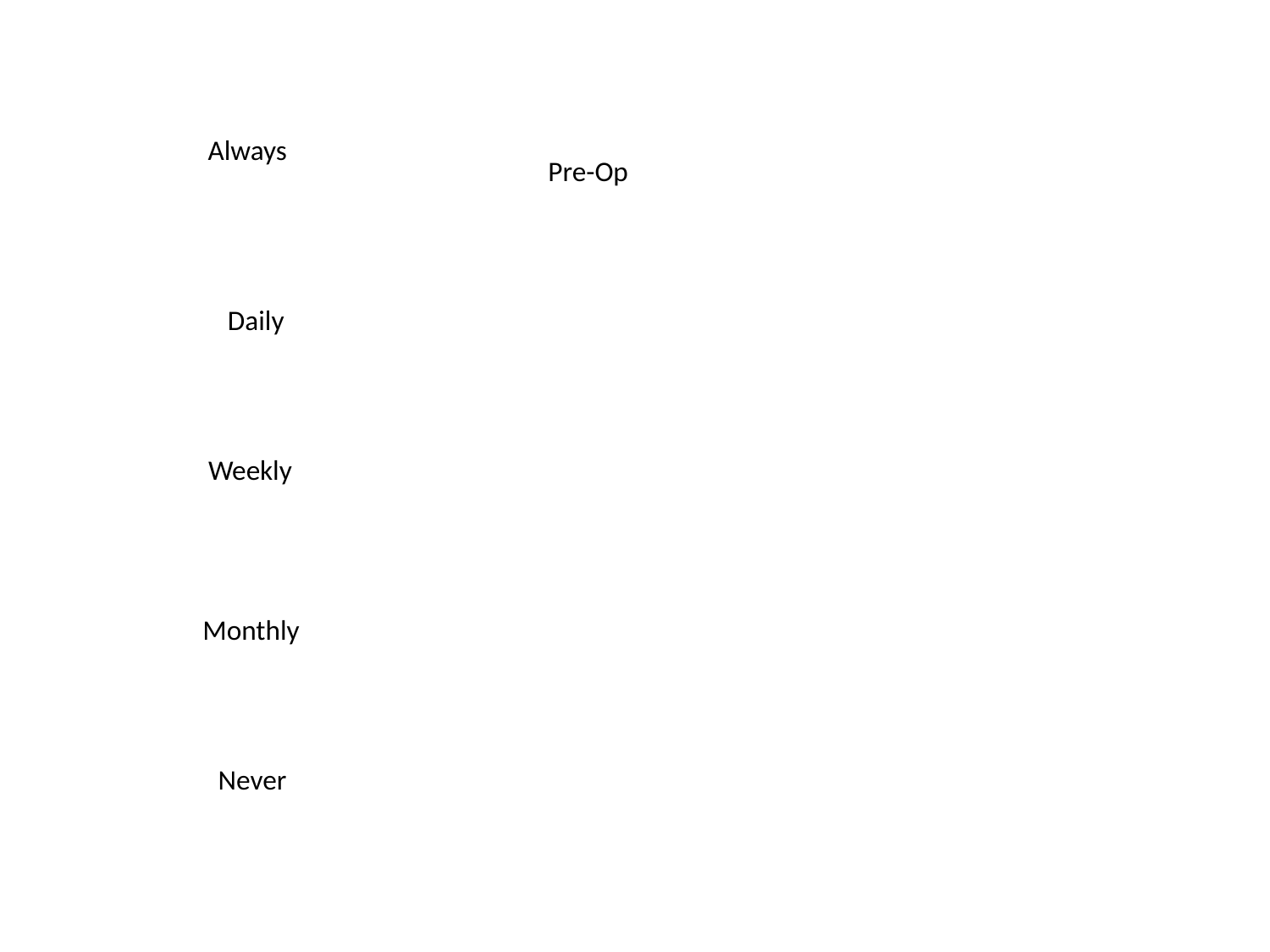

Always
Daily
Weekly
Monthly
Never
Pre-Op
