# Supplementary material for: Ultrasound Guided Arthroscopic Removal of Calcific Tendonitis: A Minimum of 2-Year Followup
Source: J Clin Med. 2023 Apr 25;12(9):3114. doi: 10.3390/jcm12093114 (PMC10179588; doi:10.3390/jcm12093114)
Supplement: Supplementary file 1 [file jcm-12-03114-s001.zip › Generic Graph Templates/Level of Activities at Work.pptx]

## Slide 1
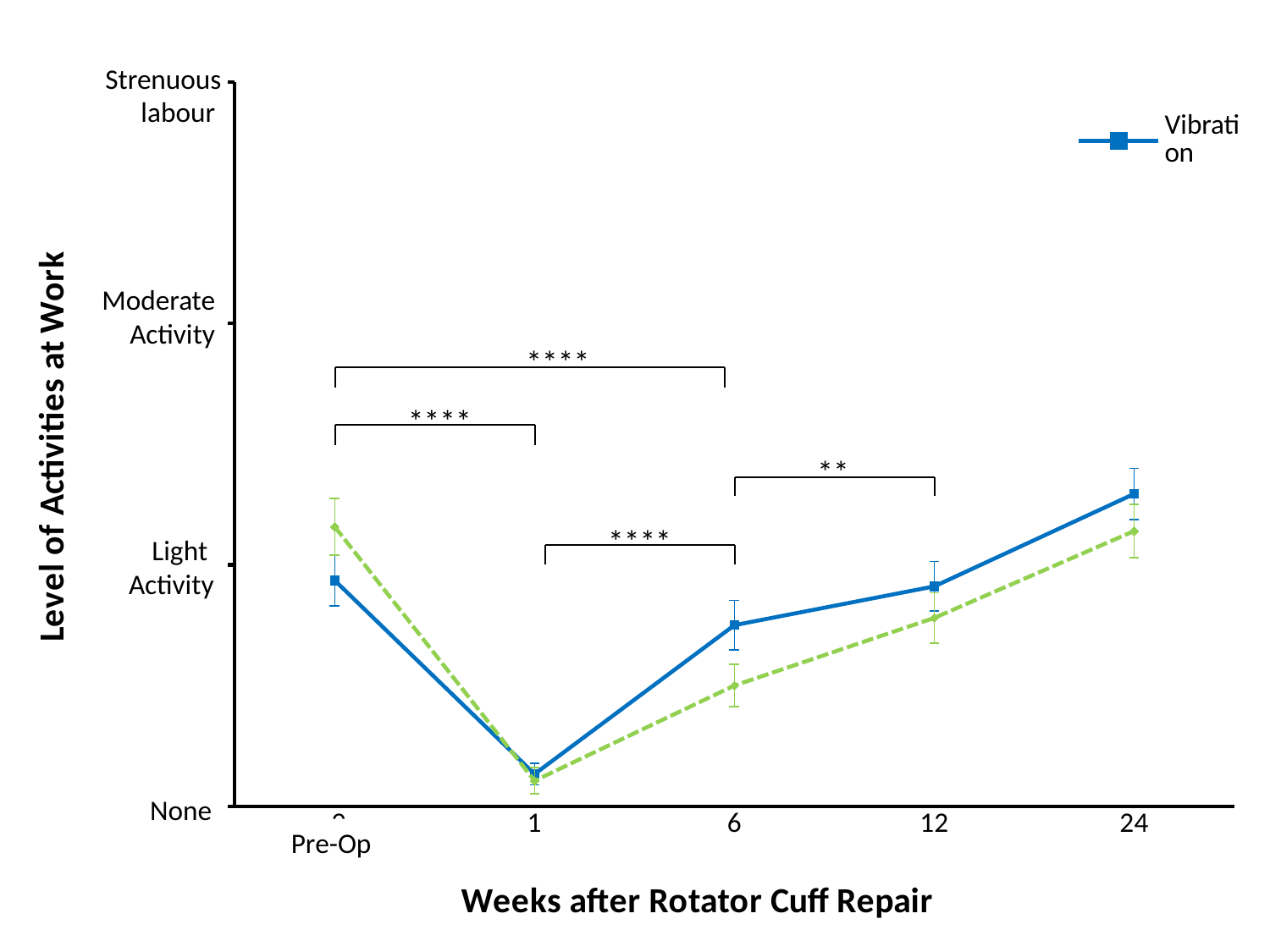

Strenuous
labour
Moderate
Activity
Light
Activity
None
### Chart
| Category | Vibration | Placebo |
|---|---|---|
| -9 | 0.9354838709677417 | 1.1578947368421049 |
| 1 | 0.1333333333333334 | 0.10526315789473686 |
| 6 | 0.7500000000000002 | 0.5 |
| 12 | 0.9107142857142856 | 0.78 |
| 24 | 1.294117647058824 | 1.1399999999999995 |****
****
**
****
Pre-Op

## Slide 2
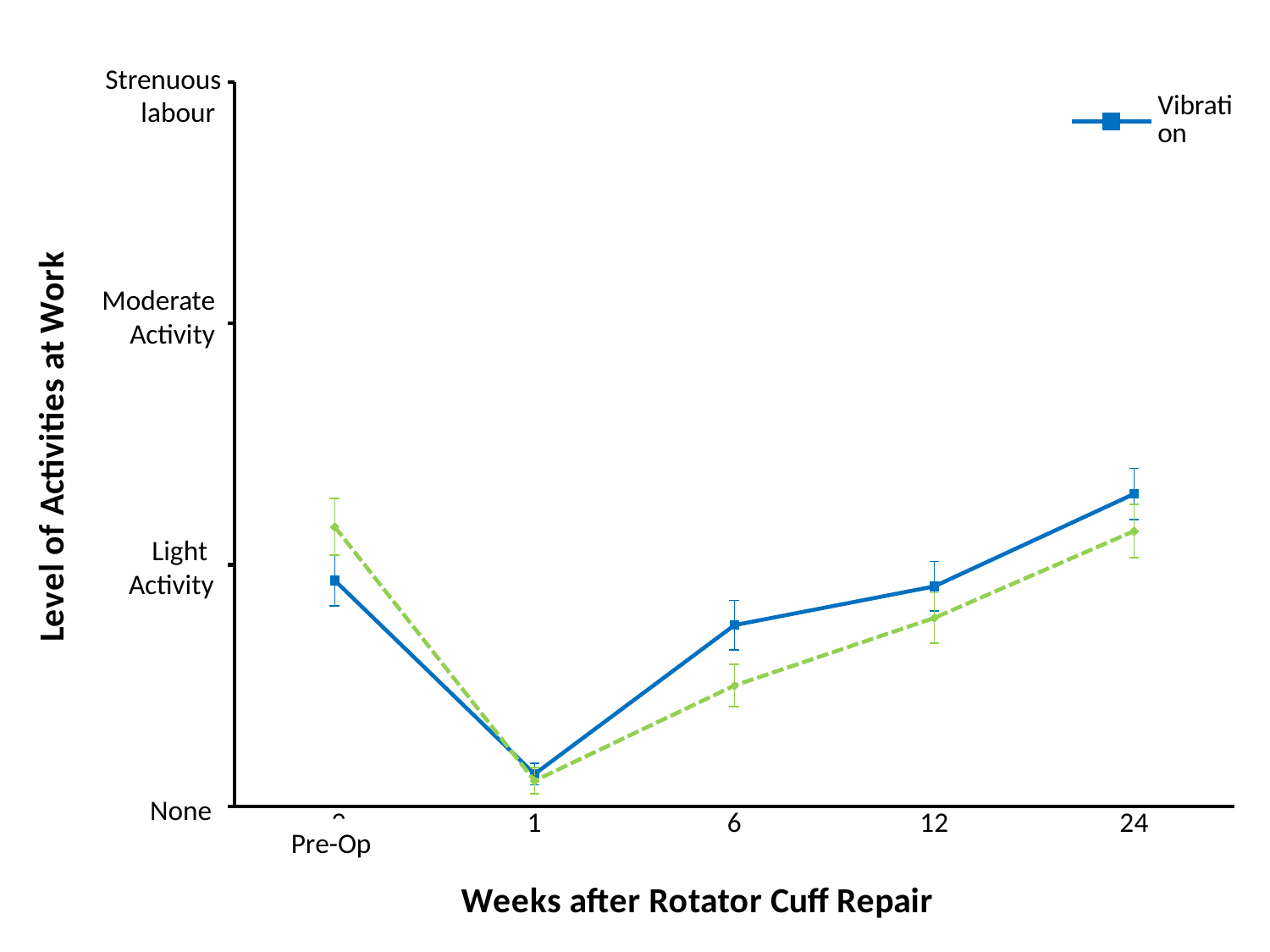

Strenuous
labour
Moderate
Activity
Light
Activity
None
### Chart
| Category | Vibration | Placebo |
|---|---|---|
| -9 | 0.9354838709677414 | 1.1578947368421044 |
| 1 | 0.13333333333333341 | 0.10526315789473686 |
| 6 | 0.7500000000000004 | 0.5 |
| 12 | 0.9107142857142856 | 0.78 |
| 24 | 1.294117647058824 | 1.139999999999999 |Pre-Op

## Slide 3
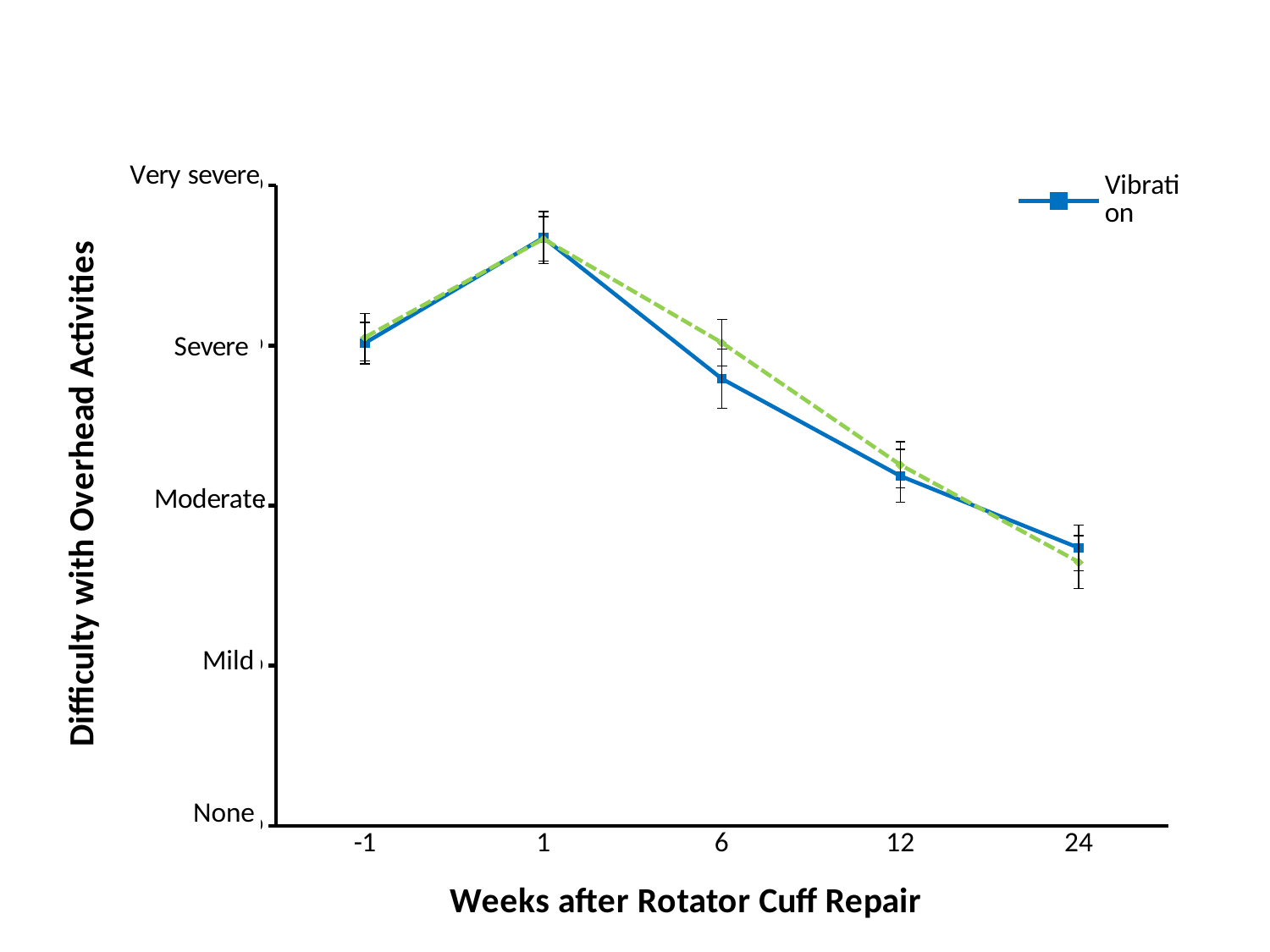

### Chart
| Category | Vibration | Placebo |
|---|---|---|
| -1 | 3.0163934426229546 | 3.051724137931035 |
| 1 | 3.6744186046511627 | 3.6666666666666665 |
| 6 | 2.7924528301886746 | 3.018181818181821 |
| 12 | 2.1851851851851847 | 2.254901960784318 |
| 24 | 1.7358490566037739 | 1.6470588235294121 |

## Slide 4
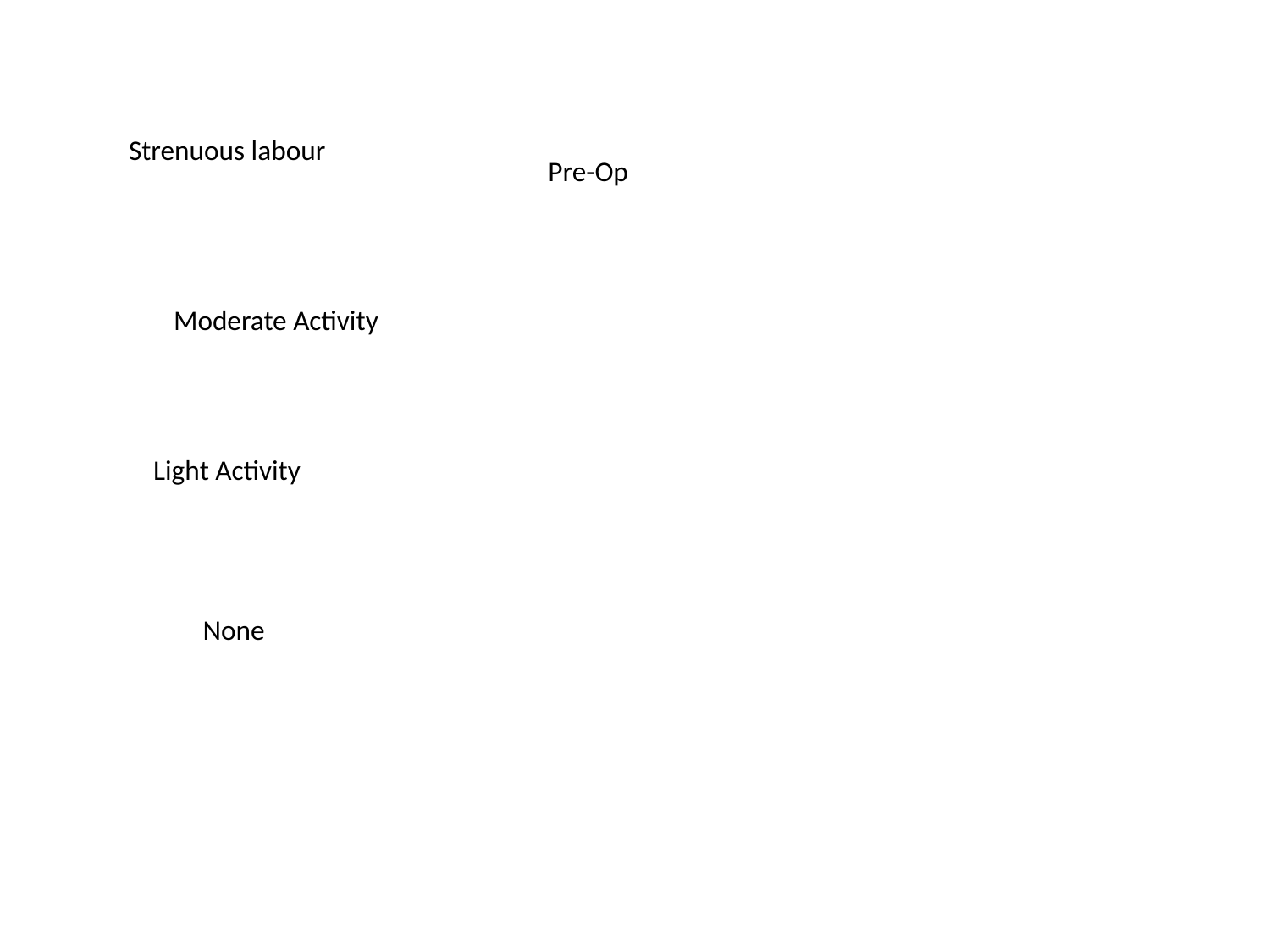

Strenuous labour
Moderate Activity
Light Activity
None
Pre-Op
