# Supplementary material for: Ultrasound Guided Arthroscopic Removal of Calcific Tendonitis: A Minimum of 2-Year Followup
Source: J Clin Med. 2023 Apr 25;12(9):3114. doi: 10.3390/jcm12093114 (PMC10179588; doi:10.3390/jcm12093114)
Supplement: Supplementary file 1 [file jcm-12-03114-s001.zip › Generic Graph Templates/Level of Shoulder Pain during Sleep.pptx]

## Slide 1
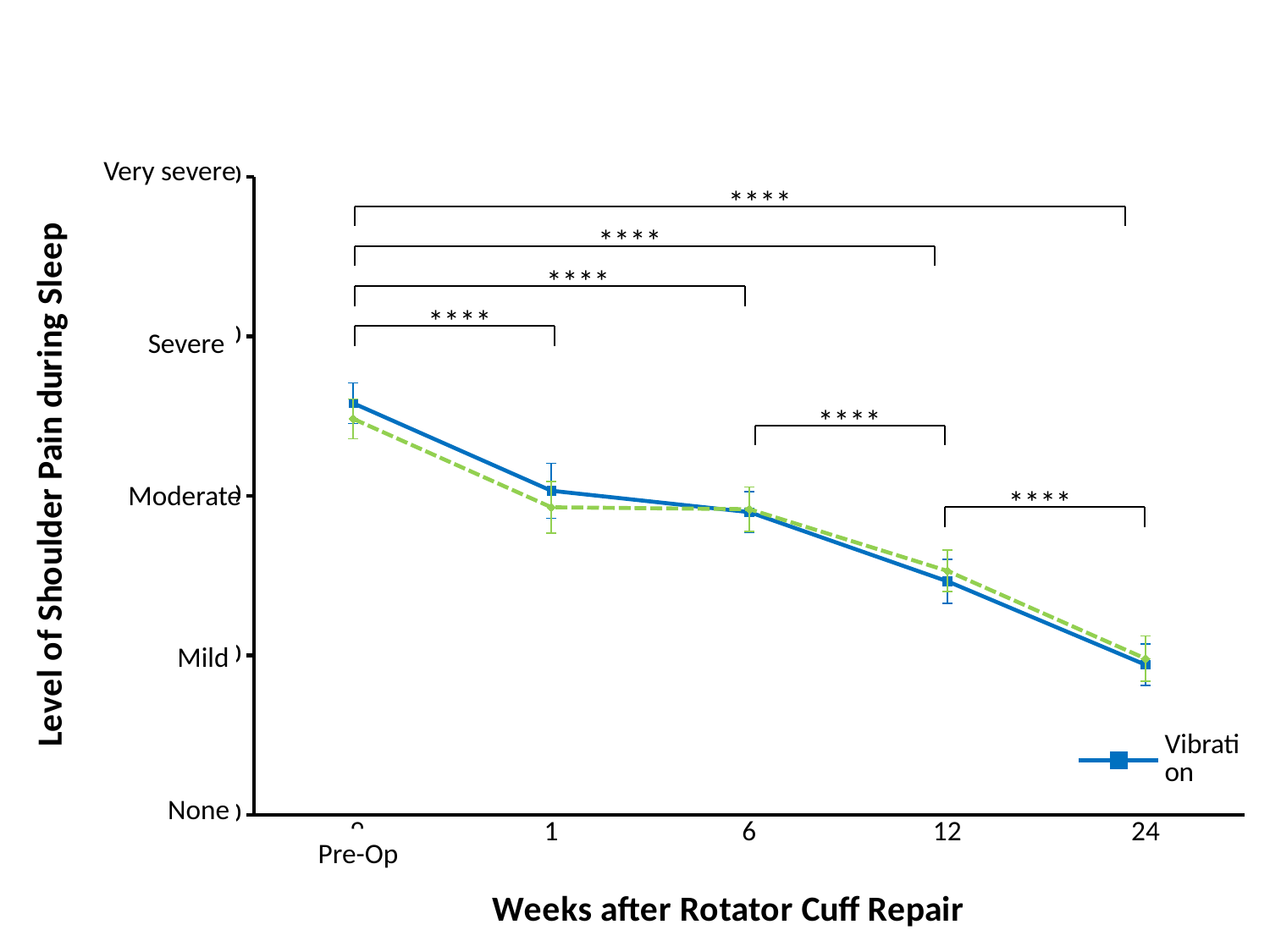

### Chart
| Category | Vibration | Placebo |
|---|---|---|
| -9 | 2.5806451612903225 | 2.4827586206896526 |
| 1 | 2.032258064516125 | 1.9285714285714293 |
| 6 | 1.8983050847457645 | 1.9166666666666663 |
| 12 | 1.4642857142857153 | 1.5294117647058822 |
| 24 | 0.9411764705882356 | 0.9795918367346946 |
Very severe
Severe
Moderate
Mild
None
****
****
****
****
****
****
Pre-Op

## Slide 2
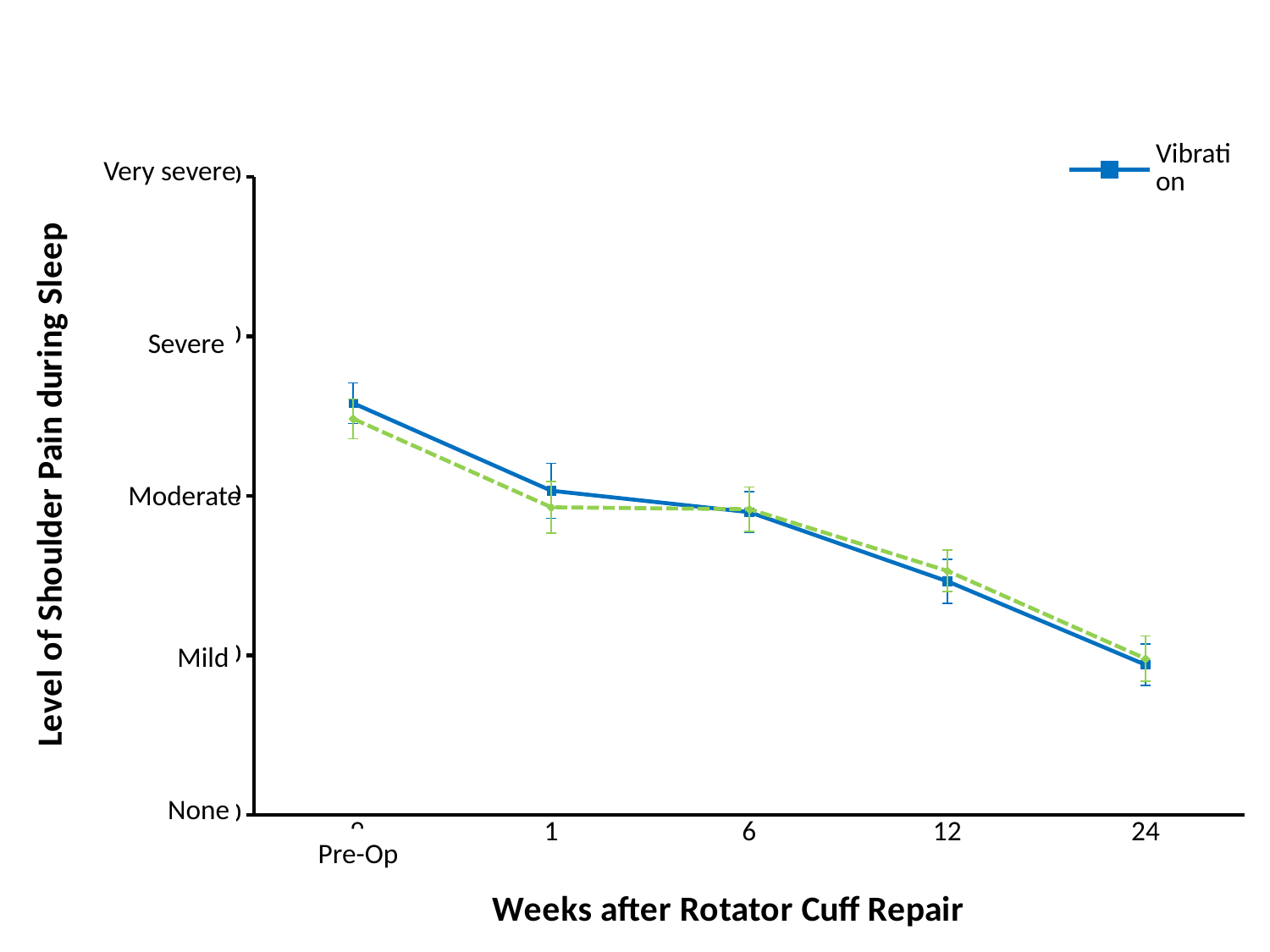

### Chart
| Category | Vibration | Placebo |
|---|---|---|
| -9 | 2.5806451612903225 | 2.4827586206896535 |
| 1 | 2.0322580645161263 | 1.928571428571429 |
| 6 | 1.898305084745764 | 1.9166666666666665 |
| 12 | 1.4642857142857149 | 1.5294117647058822 |
| 24 | 0.9411764705882356 | 0.9795918367346943 |
Very severe
Severe
Moderate
Mild
None
Pre-Op

## Slide 3
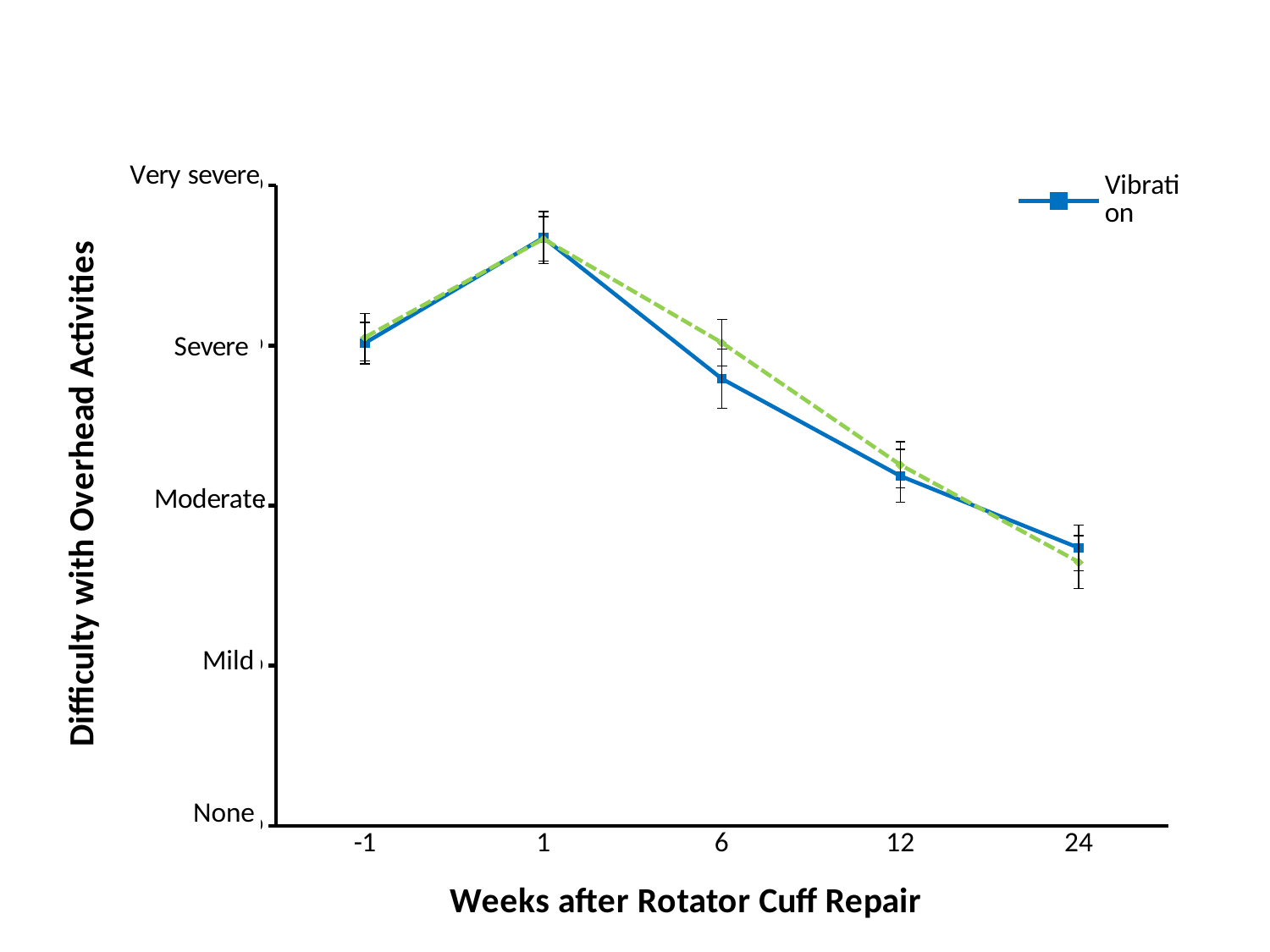

### Chart
| Category | Vibration | Placebo |
|---|---|---|
| -1 | 3.016393442622955 | 3.051724137931035 |
| 1 | 3.6744186046511627 | 3.6666666666666665 |
| 6 | 2.7924528301886737 | 3.018181818181821 |
| 12 | 2.1851851851851847 | 2.2549019607843186 |
| 24 | 1.7358490566037739 | 1.6470588235294121 |

## Slide 4
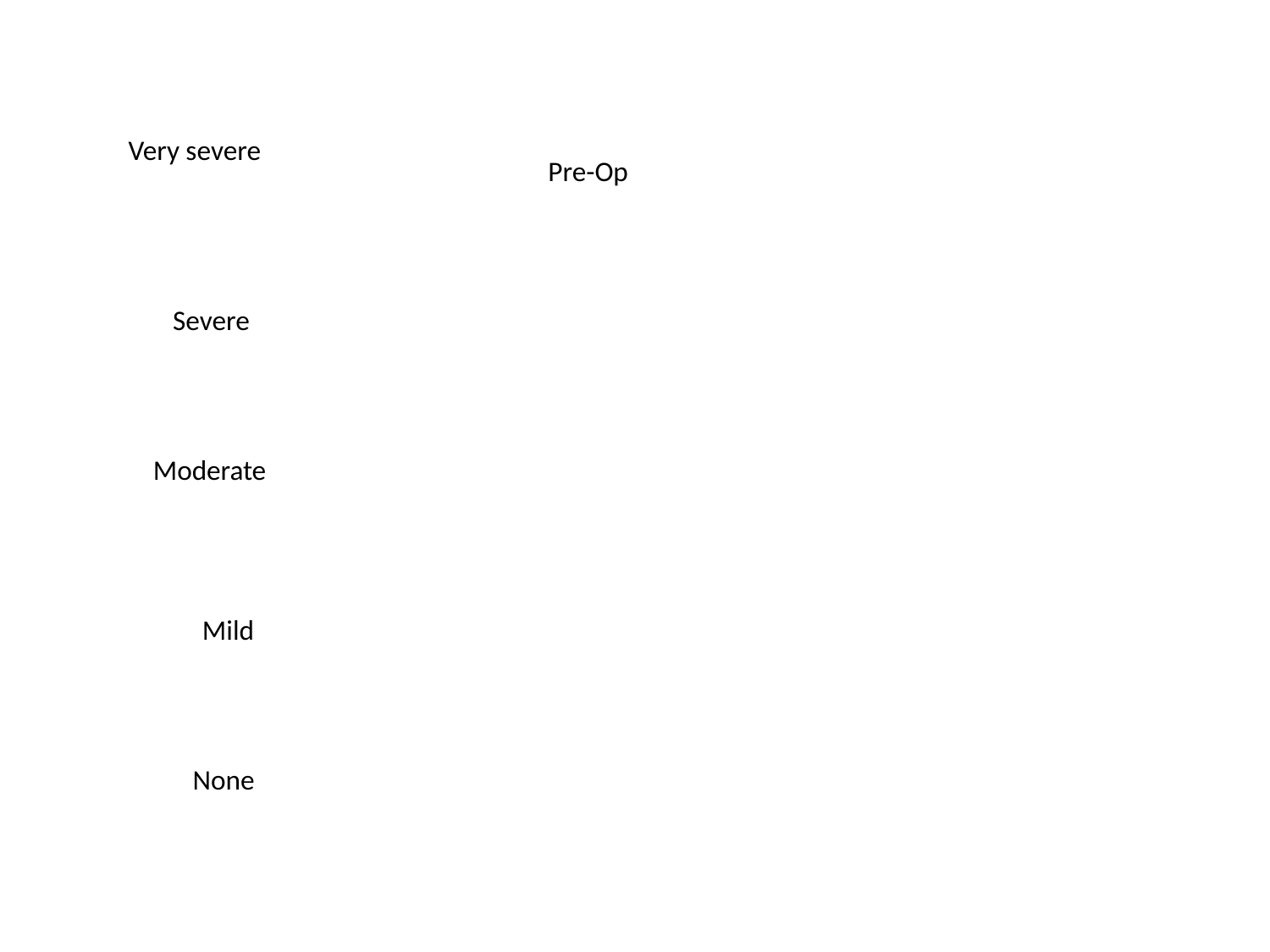

Very severe
Severe
Moderate
Mild
None
Pre-Op
