# Supplementary material for: Ultrasound Guided Arthroscopic Removal of Calcific Tendonitis: A Minimum of 2-Year Followup
Source: J Clin Med. 2023 Apr 25;12(9):3114. doi: 10.3390/jcm12093114 (PMC10179588; doi:10.3390/jcm12093114)
Supplement: Supplementary file 1 [file jcm-12-03114-s001.zip › Generic Graph Templates/Level of Shoulder Stiffness.pptx]

## Slide 1
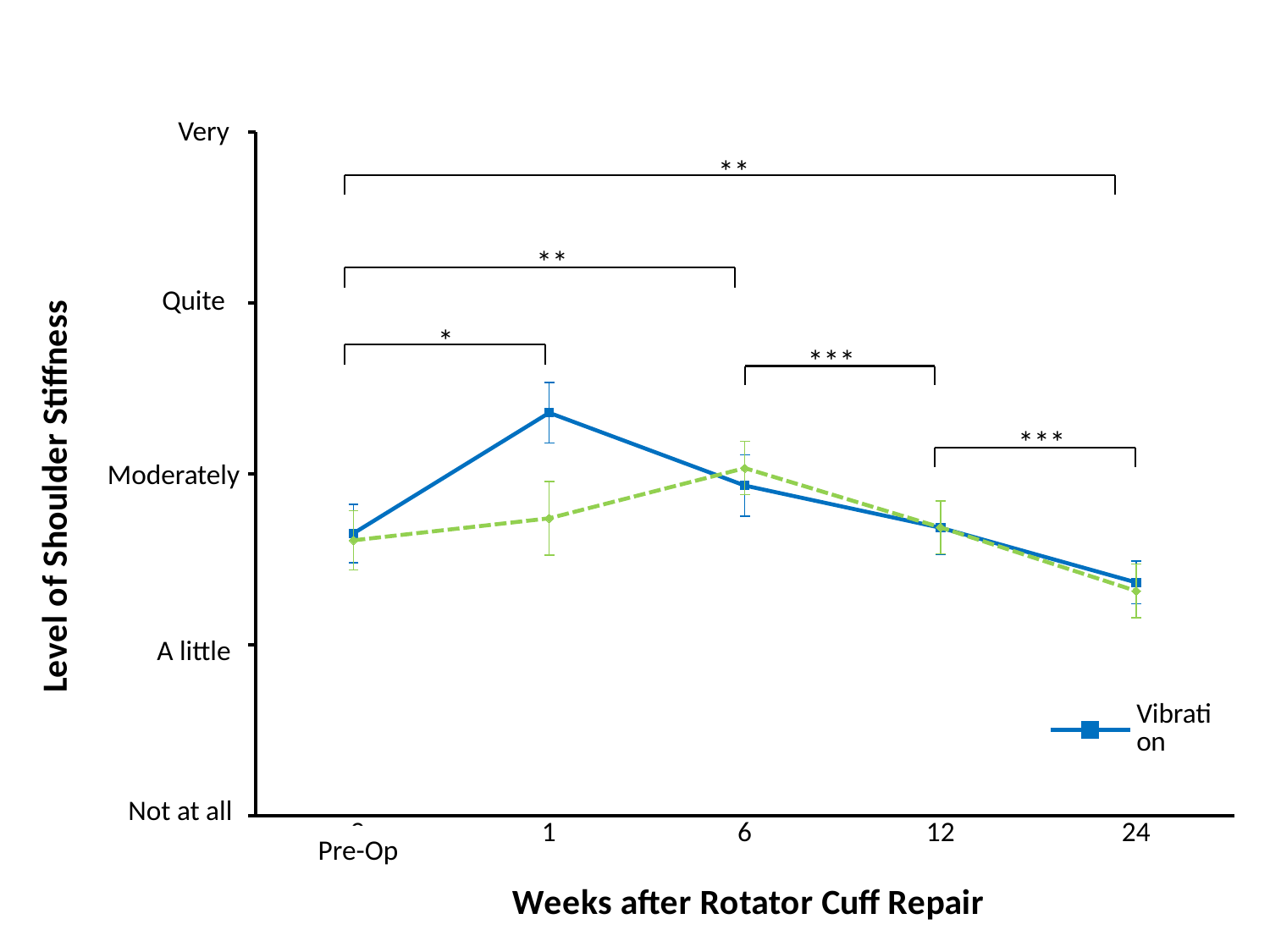

Very
Quite
Moderately
A little
Not at all
### Chart
| Category | Vibration | Placebo |
|---|---|---|
| -9 | 1.6507936507936503 | 1.6101694915254237 |
| 1 | 2.357142857142857 | 1.739130434782609 |
| 6 | 1.9310344827586203 | 2.033333333333333 |
| 12 | 1.6842105263157905 | 1.6862745098039222 |
| 24 | 1.3636363636363635 | 1.313725490196078 |**
**
*
***
***
Pre-Op

## Slide 2
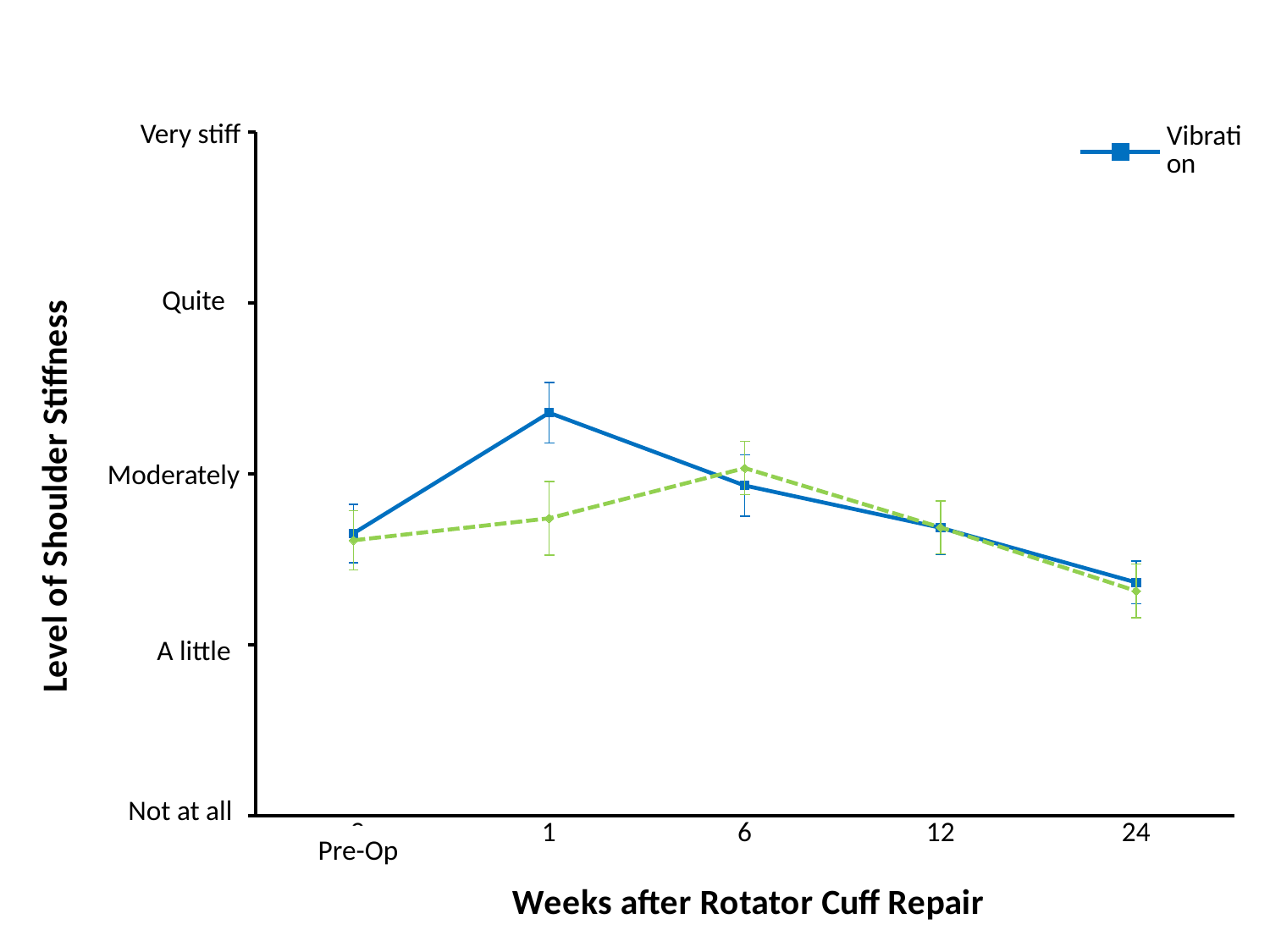

Very stiff
Quite
Moderately
A little
Not at all
### Chart
| Category | Vibration | Placebo |
|---|---|---|
| -9 | 1.6507936507936498 | 1.6101694915254237 |
| 1 | 2.357142857142857 | 1.7391304347826089 |
| 6 | 1.9310344827586206 | 2.033333333333333 |
| 12 | 1.6842105263157914 | 1.6862745098039227 |
| 24 | 1.3636363636363635 | 1.3137254901960778 |Pre-Op

## Slide 3
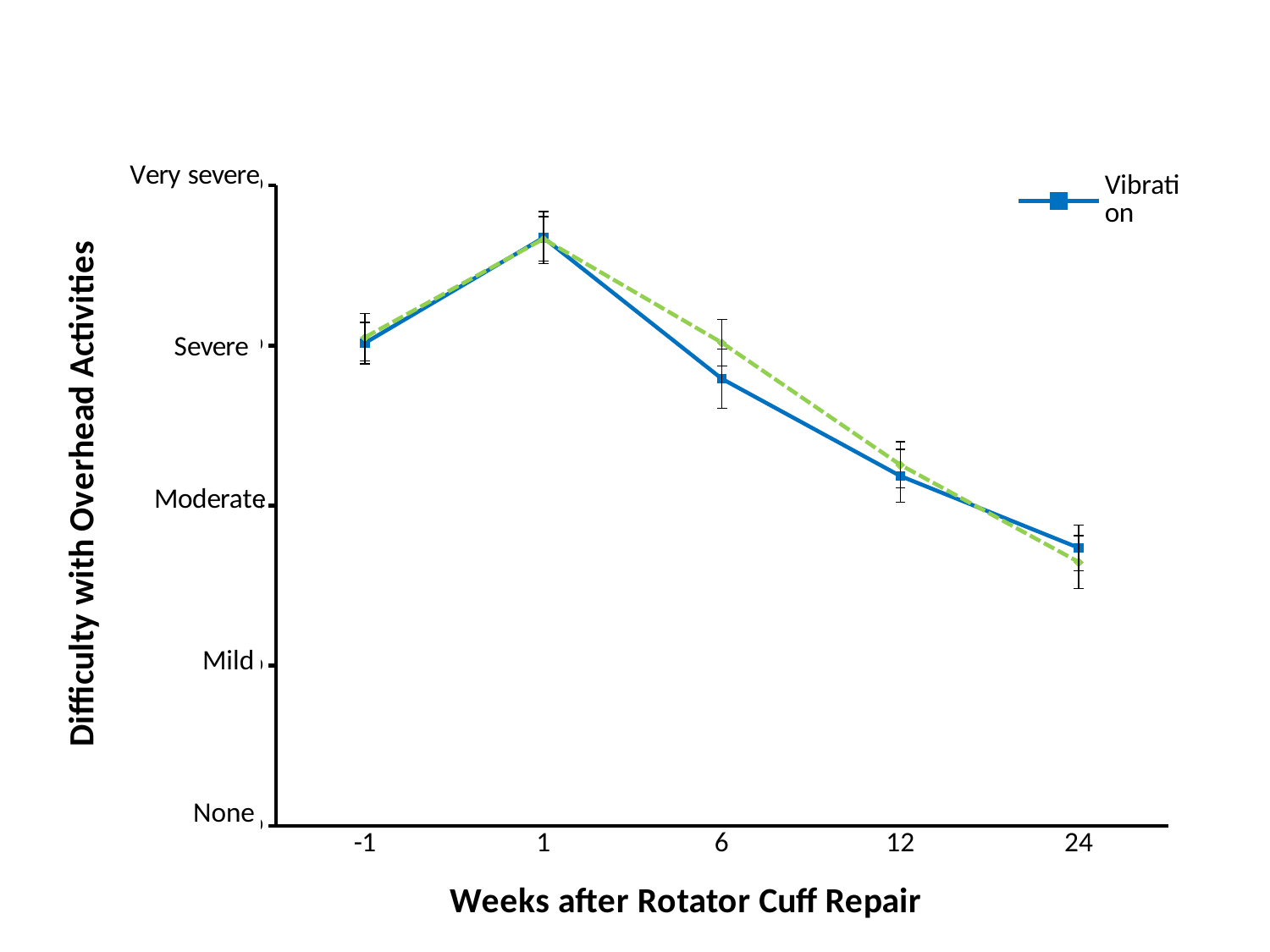

### Chart
| Category | Vibration | Placebo |
|---|---|---|
| -1 | 3.0163934426229546 | 3.051724137931035 |
| 1 | 3.6744186046511627 | 3.6666666666666665 |
| 6 | 2.7924528301886746 | 3.018181818181821 |
| 12 | 2.1851851851851847 | 2.254901960784318 |
| 24 | 1.7358490566037739 | 1.6470588235294121 |

## Slide 4
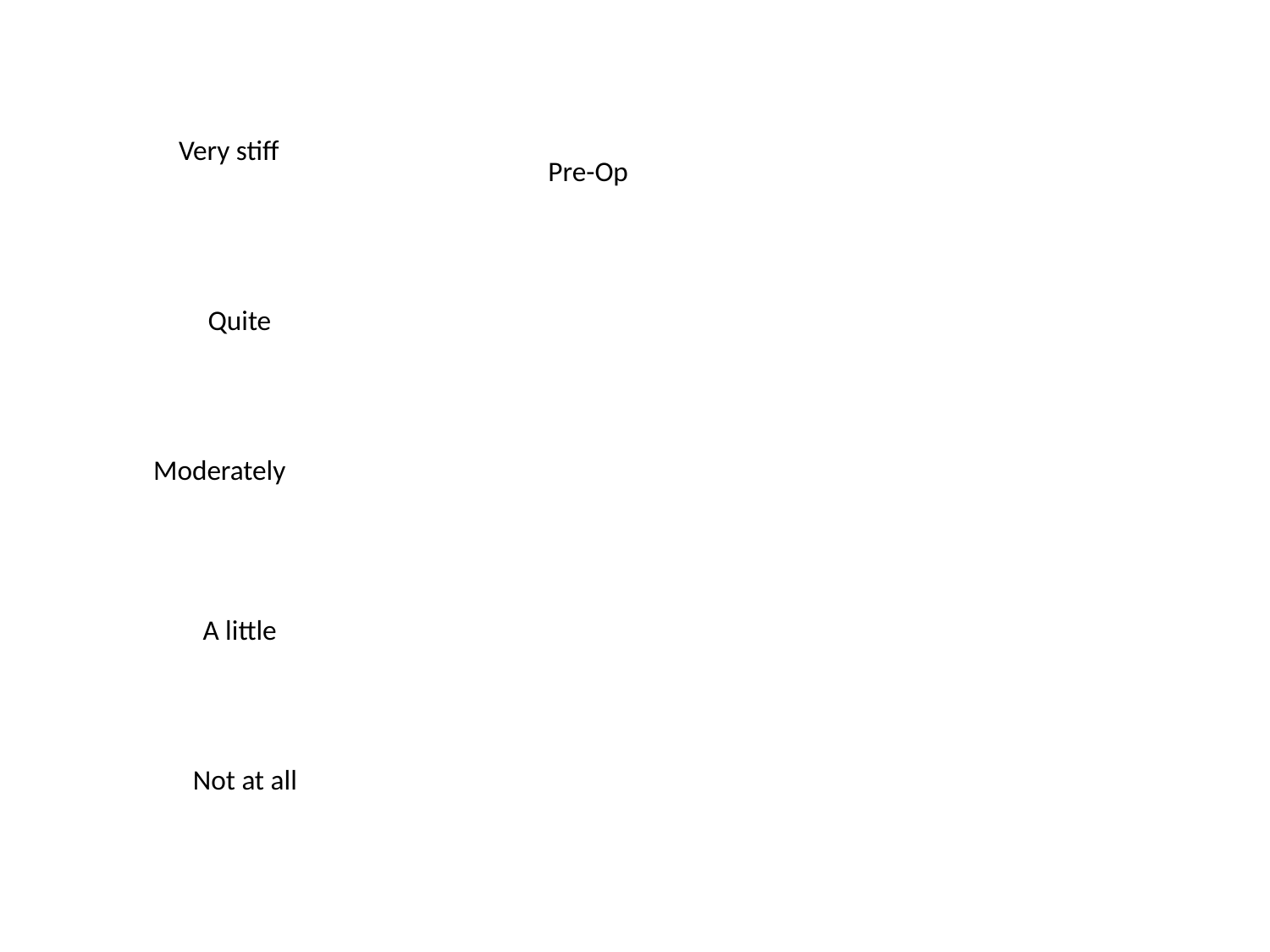

Very stiff
Quite
Moderately
A little
Not at all
Pre-Op
