# Supplementary material for: Ultrasound Guided Arthroscopic Removal of Calcific Tendonitis: A Minimum of 2-Year Followup
Source: J Clin Med. 2023 Apr 25;12(9):3114. doi: 10.3390/jcm12093114 (PMC10179588; doi:10.3390/jcm12093114)
Supplement: Supplementary file 1 [file jcm-12-03114-s001.zip › Generic Graph Templates/ROM IR.pptx]

## Slide 1
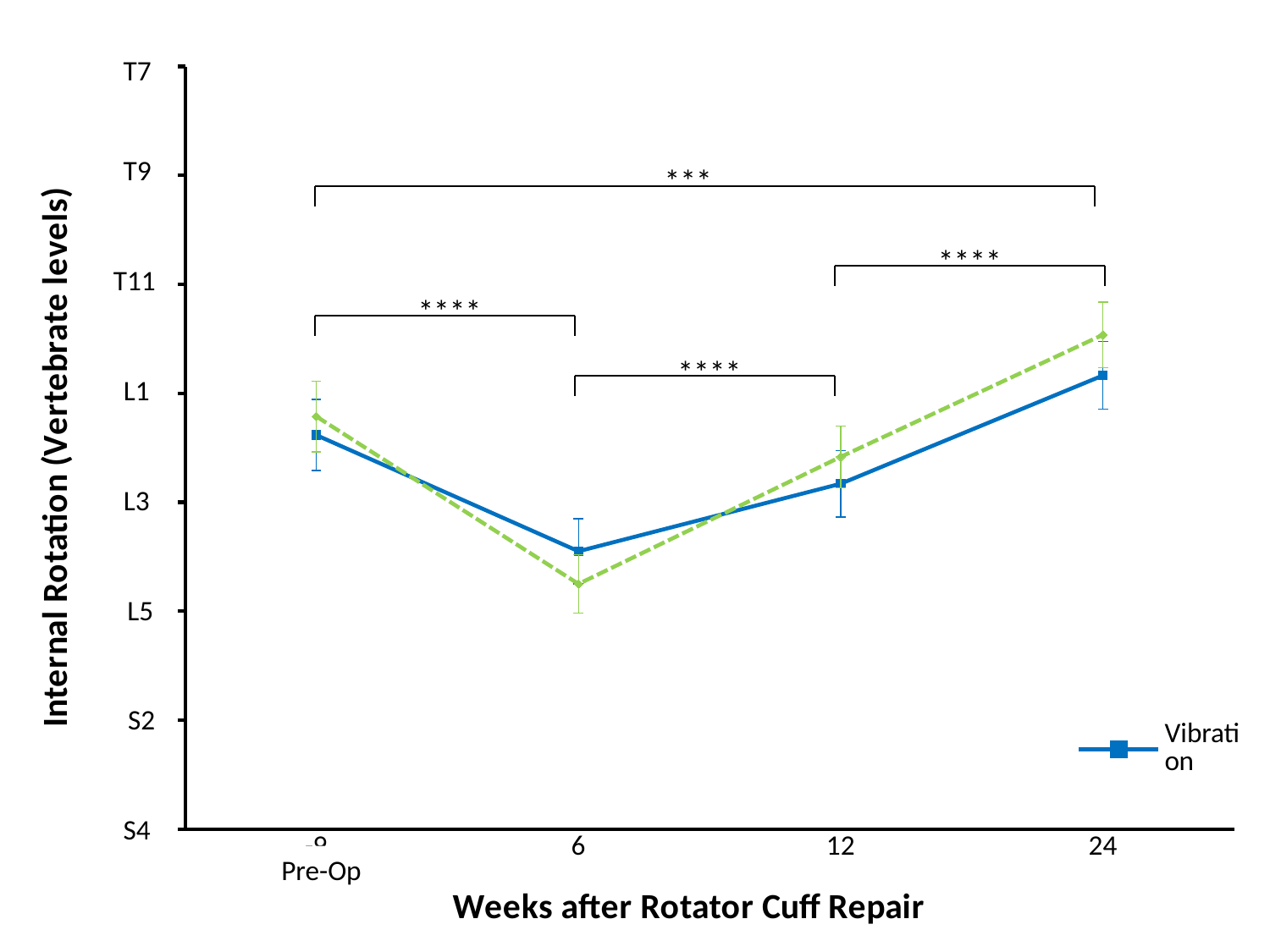

### Chart
| Category | Vibration | Placebo |
|---|---|---|
| -9 | 8.233333333333325 | 8.57627118644067 |
| 6 | 6.1 | 5.5 |
| 12 | 7.339285714285714 | 7.833333333333338 |
| 24 | 9.327586206896559 | 10.074074074074073 |
T7
T11
L3
L5
S4
T9
L1
S2
***
****
****
****
Pre-Op

## Slide 2
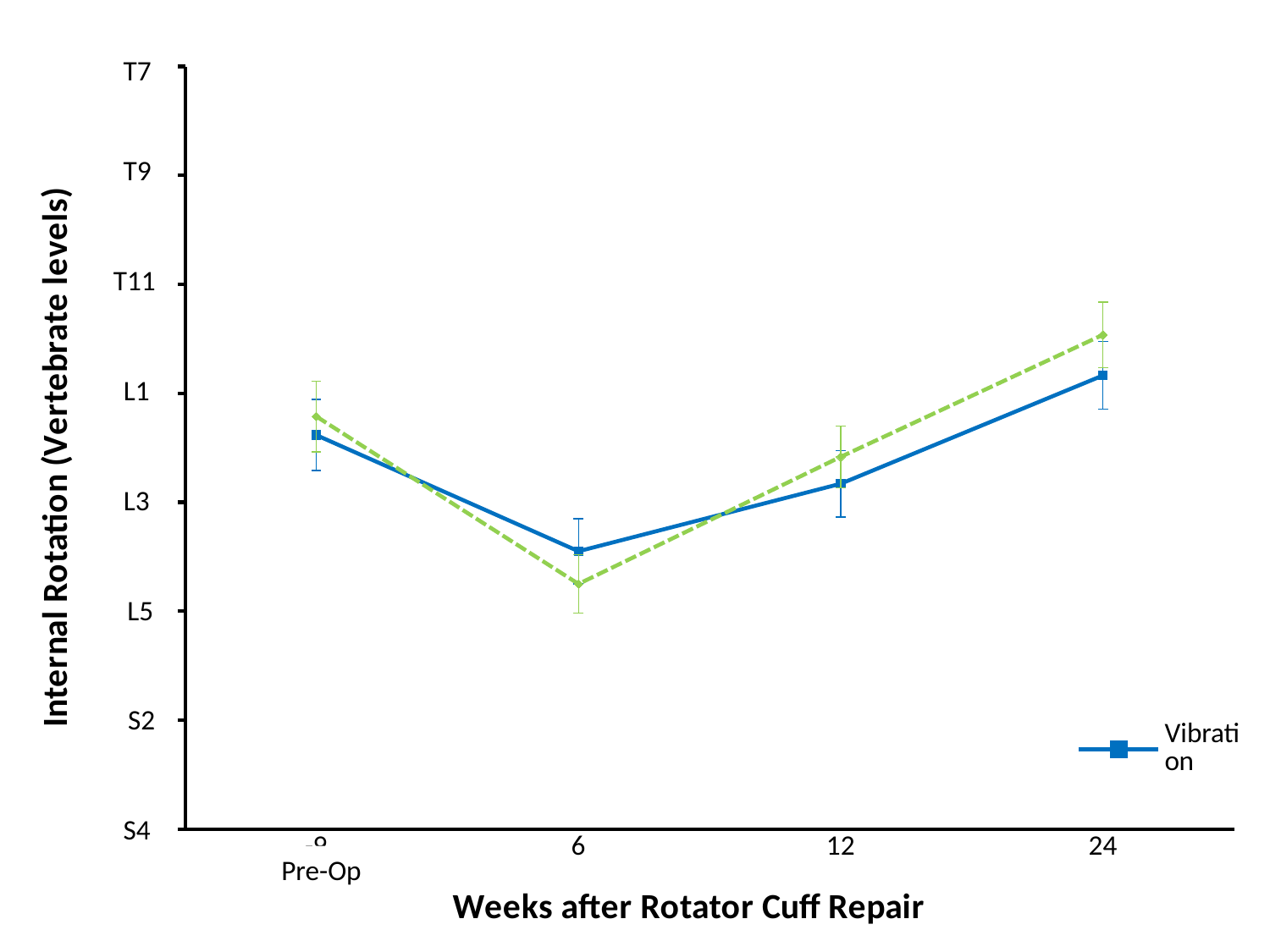

### Chart
| Category | Vibration | Placebo |
|---|---|---|
| -9 | 8.233333333333322 | 8.576271186440666 |
| 6 | 6.1 | 5.5 |
| 12 | 7.339285714285714 | 7.83333333333334 |
| 24 | 9.327586206896562 | 10.074074074074073 |
T7
T11
L3
L5
S4
T9
L1
S2
Pre-Op

## Slide 3
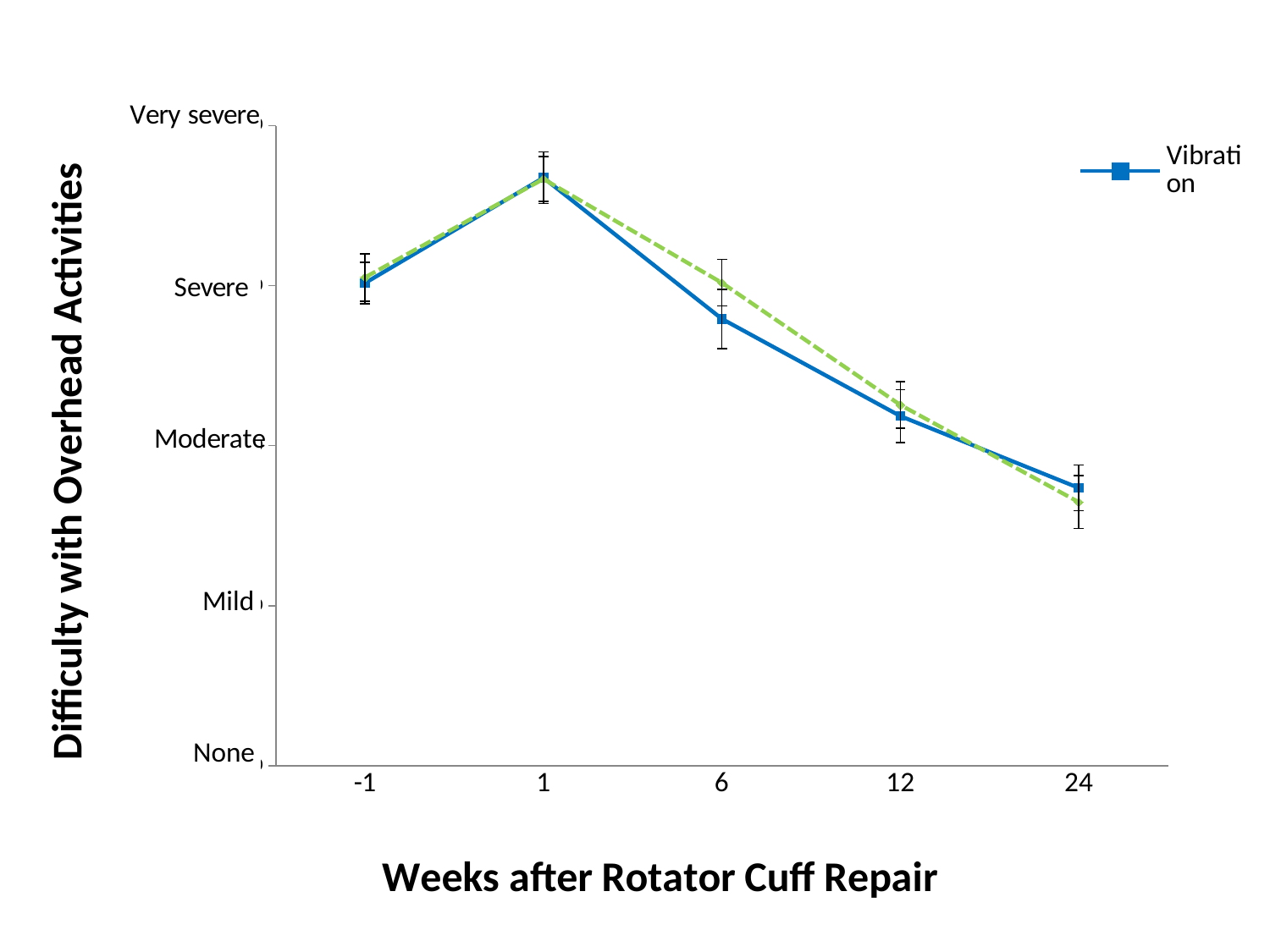

### Chart
| Category | Vibration | Placebo |
|---|---|---|
| -1 | 3.016393442622954 | 3.051724137931035 |
| 1 | 3.6744186046511627 | 3.6666666666666665 |
| 6 | 2.7924528301886755 | 3.0181818181818207 |
| 12 | 2.1851851851851847 | 2.2549019607843173 |
| 24 | 1.7358490566037739 | 1.6470588235294121 |

## Slide 4
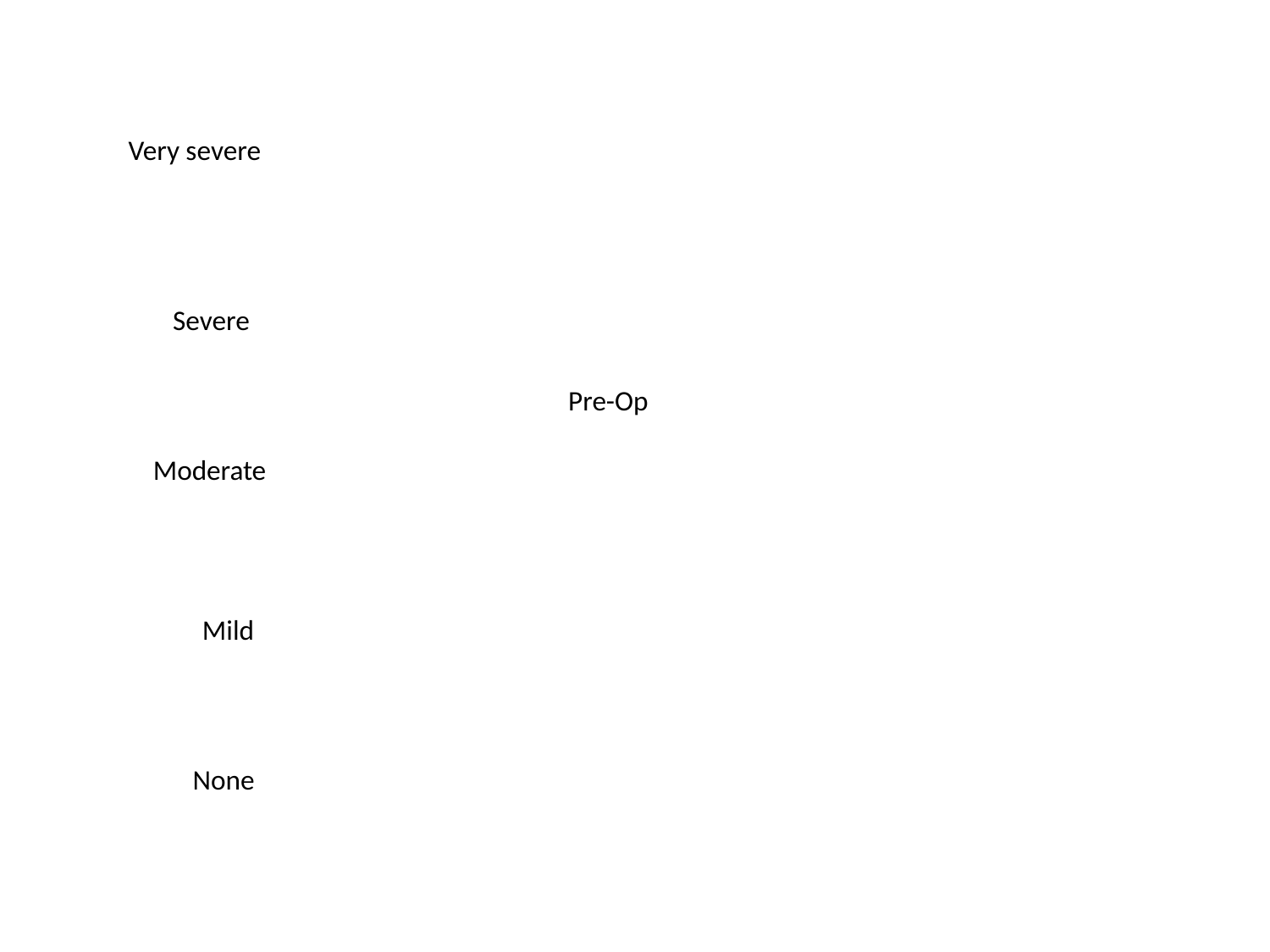

Very severe
Severe
Moderate
Mild
None
Pre-Op
